# A novel ANN-based approach for fault detection and classification in modern TCSC-compensated transmission lines integrated with DFIG-based wind farms utilizing WST

**DOI:** 10.1038/s41598-026-51960-7

**Published:** 2026-05-20

**Authors:** Eyad S. Oda, Abdelrahman Mamdouh M. Habib, Basem E. Elnaghi, Naema M. Mansour

**Affiliations:** Department of Electrical Engineering, Faculty of Engineering, Suez Canal University, Ismailia, 41522 Egypt

**Keywords:** Fault detection, Fault classification, DWT, WST, ANN, DFIG, TCSC, Sub-synchronous resonance

## Abstract

The dynamic characteristics of doubly fed induction generator (DFIG)-based wind farms, together with the variable reactance introduced by a thyristor-controlled series capacitor (TCSC) compensation, significantly alter fault current profiles and apparent line impedance, which may lead to maloperation of conventional transmission line protection schemes. In this paper, an intelligent fault detection and classification approach for a TCSC-compensated transmission line integrated with a DFIG-based wind power generation system is proposed. The method used the Wavelet Scattering Transform (WST) for robust feature extraction with a feed-forward back propagation neural network (BPNN) for accurate classification. The proposed approach exploits the inherent stability and invariance properties of WST to extract discriminative features from transmission line current signals under dynamic operating conditions. Its performance is evaluated through extensive simulation studies involving 3240 fault cases covering variations in fault type, location, inception angle, wind-farm operating conditions, and TCSC compensation levels. A comparative analysis with discrete wavelet transform (DWT)-based features is conducted in terms of detection latency, robustness, computational efficiency, and classification accuracy. Simulation results demonstrate that the WST-based approach outperforms conventional DWT-based methods, achieving high classification accuracy up to 100% with strong robustness to noise and operating variations, while maintaining a response time suitable for practical protection applications. These results confirm the effectiveness of the proposed scheme for modern series-compensated transmission systems with high renewable energy penetration. All simulations are carried out using MATLAB.

## Introduction

The increasing penetration of renewable energy sources, particularly DFIG-based wind farms, along with the deployment of TCSCs for power flow control, has introduced significant challenges to the reliable operation of transmission line protection systems. Modifications in fault current characteristics and apparent line impedance have emerged. Moreover, the rapid switching of thyristors introduces high-frequency and nonlinear components into system currents during fault events making traditional detection techniques prone to misclassification or delayed operation^1–3^. Thereby challenging the reliability and accuracy of conventional transmission line protection schemes. Consequently, fast and accurate fault detection and classification are critical to ensuring system protection, post-fault recovery, and the reliable operation of both conventional grids and renewable energy resources^4,5^. Accurate discrimination between different fault types thus requires advanced signal-processing algorithms that can extract meaningful time–frequency features from highly dynamic transients^4,6^. Although DFIG-based wind farms and TCSC-compensated transmission lines are increasingly integrated in modern power systems, relatively few studies have addressed the associated protection challenges under this combined scenario. Most existing works consider wind-farm integration or FACTS-based compensation independently, without fully accounting for their combined impact and interactions^1–3,7^. The majority of the proposed approaches need current measurements at both ends of the line, which necessitate a communication network, making them practically less desirable^2,3,7^. In order to classify the fault of the compensated transmission line using the IEEE first benchmark power system, the DWT and ANN-based algorithm was introduced in^4^. This method uses a transmission line lumped parameter model, which is a highly simplified model rather than a fully practical distributed parameters model that is taken into consideration in our study. Furthermore, its reliance on the threshold values as in^2,8,9^ at which faulty and nonfaulty cases can be distinguished poses significant issues due to variations in wind speed, TCSC compensation level, and fault impedance.

In^10^, the authors proposed a novel protection scheme based on ANN and Support Vector Machine (SVM). Although the reported accuracy reached approximately 99%, the proposed method requires voltage and current measurements from both ends of the transmission that necessitates a reliable communication infrastructure and synchronized data acquisition, which increases system complexity and implementation cost. Furthermore, the required re-scaling and preprocessing of measured signals to construct the training dataset introduce additional computational burden. The classification approach introduced in^11^ achieved recorded accuracy of 99.82% using a dataset composed of voltage and current measured at the relaying point. While this result demonstrates strong classification capability, the method primarily relies on time-domain signals. However, critical discriminative features are often embedded within the transient characteristics of these signals and may not be fully exploited through conventional time-domain analysis.

With accuracy 100% for fault detection and 99.85% fault classification, the normalized DWT details and approximation coefficients of the measured voltage, voltage-angle, current, current-angle, and frequency signals data set training Weighted Extreme Learning Machine (WELM) in^12^. Despite reasonable classification accuracy, DWT-based algorithms remain sensitive to fault inception angle, sampling frequency, and the appropriate decomposition level selection for feature extraction that significantly influences the extracted wavelet coefficients, thereby affecting the reliability and consistency of the classification results. The majority of fault classification methods documented in the literature use feature matrices that are obtained from the DWT^13–20^**.** However, a number of crucial design factors, such as the number of decomposition levels, the mother wavelet selection, and the sampling frequency, are not standardized in these approaches. The lack of standardized criteria for these characteristics causes performance variability and restricts how broadly the suggested approaches can be applied. Additionally, the fault initiation angle has a major impact on the transient signal characteristics and, in turn, the classification accuracy, as the derived wavelet coefficients are quite sensitive to it, especially at higher decomposition levels. Most of these schemes exhibit notable limitations, including the requirement for an impractically high sampling rate and increased sensitivity to system noise due to its dependence on high-frequency signal components. These limitations emphasize the necessity for more robust, noise insensitive, and time-invariant feature extraction tools to ensure reliable fault classification in modern power systems. In this context, the WST emerges as a powerful and robust analytical tool^21^. Unlike conventional DWT, WST provides stable and time-invariant representations of transient signals while preserving essential time–frequency information. Its inherent resistance to noise and reduced sensitivity to signal distortions make it particularly suitable for analyzing nonlinear and highly dynamic power system disturbances. Therefore, WST offers a reliable framework for discriminative features extraction that enhance classification accuracy and improve overall protection performance under varying operating conditions. Although DWT has been widely adopted for power system fault detection, the application of WST in this domain remains largely unexplored^21,22^, despite its demonstrated superiority in feature extraction and translation invariance in image and audio classification tasks^23,24^. In this study, the WST is introduced for the first time for fault detection and classification in TCSC-compensated transmission lines integrated with high wind energy penetration. The proposed approach utilizes WST to effectively track the various dynamic variations inherent in TCSC-compensated lines, such as changes in compensation level, wind speed fluctuations, and nonlinear fault transients. By capturing stable and discriminative time–frequency representations of only the measured 3-phase and the ground fault current signals, an intelligent fault detection and classification algorithm based on the first three-level WST coefficients feature matrix that is trained with ANN is presented in this study. The first-level DWT detail coefficients (cD1) and second-level detail coefficients (cD2) are utilized to construct two alternative training matrices for comparative analysis. The comparison evaluates response time, classification accuracy, and computational complexity, with the objective of identifying the most efficient approach. The main contributions of this study are summarized as follows:

- A novel and reliable protection framework is developed for transmission lines in a complex environment involving TCSC-compensated networks integrated with DFIG-based wind farms, addressing the challenges introduced by renewable energy variability and series compensation.
- The WST is employed for robust feature extraction from transmission line current signals that provides stable, translation-invariant, and noise-robust representations under varying operating conditions***.***
- A compact and discriminative feature matrix is constructed using selected WST path coefficients, significantly reducing feature dimensionality while preserving fault-relevant information.
- A BPNN is developed for accurate classification of different fault types under different system conditions, including variations in compensation factor, fault location, resistance, inception angle, and wind-farm operating states.
- The proposed method demonstrates superior robustness compared to conventional DWT-based approaches, particularly under low fault inception angle, noise contamination, and dynamic operating scenarios.
- The effectiveness of the proposed approach is further demonstrated by achieving high classification accuracy up to 100% under varying TCSC compensation levels and wind-speed conditions. This performance is validated using a dataset of 3240 fault cases based on four current signals measured at the grid-side relaying point, confirming its suitability for modern renewable-integrated transmission systems.

The paper is structured so that the system modeling under study is described in Section II. Section III describes the suggested intelligent methodology. Section IV discusses the results. In Section V, the results are finally concluded.

## Power system model description

**Fig. 1:**
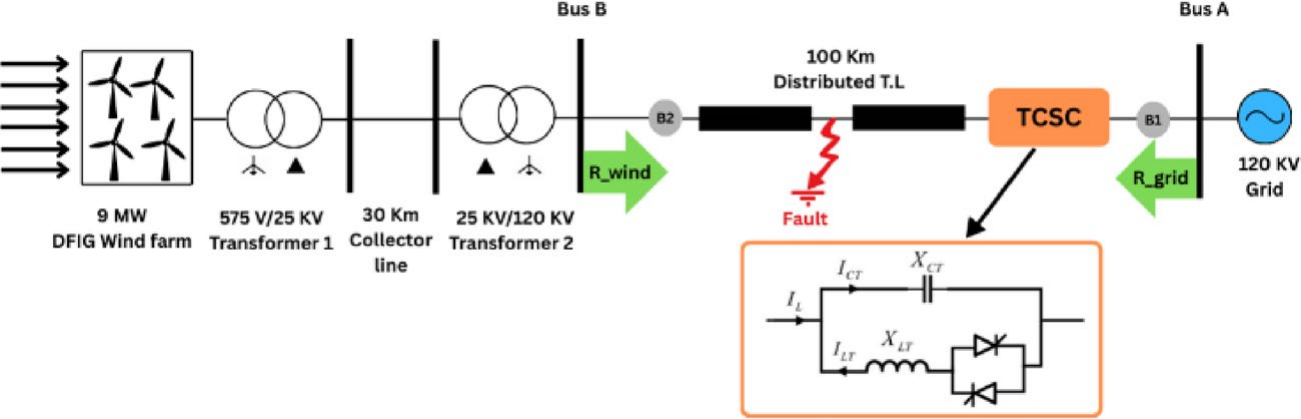
Schematic diagram of the studied TCSC-compensated transmission network with integrated DFIG-based wind farm.

The studied system is modeled and simulated using the MATLAB/Simulink environment. The system consists of a double-source transmission line integrated with a DFIG-based wind farm and compensated by a TCSC, as illustrated in Figure 1.

The transmission line (TL) is a 100 km, 120 kV, 50 Hz line modeled using a distributed-parameter representation, which provides improved accuracy for representing the line and provides higher accuracy compared to lumped models, particularly for transient and fault analysis. The electrical parameters, including per-unit-length resistance (R), inductance (L), and capacitance (C), are summarized in Table 1.

**Table 1:** Electrical parameter of distributed parameters TL model.

Parameters	unit	+ ve sequence components	-ve sequence components	Zero-sequence
R	Ω/Km	5.90 × 10^–2^	5.90 × 10^–2^	5.90 × 10^–1^
L	mH/Km	1.33	1.33	3.98
C	nF/km	9.83	9.83	5.90

The sending-end 120Kv grid (bus A) is represented by an infinite bus modeled using a the three-phase programmable voltage source with zero-impedance coupled with a three-phase impedance with mutual coupling between phases.

The receiving-end source (bus B) consists of a 575 V DFIG-based wind farm connected to a 25 kV, 5 km collector feeder through a 575 V/25 kV step-up transformer. The wind farm is further interfaced to the transmission system via a second step-up transformer rated at 25 kV/120 kV, as illustrated in Figure 1. The electrical parameters of the wind farm are summarized in Table 2.

**Table 2:** Electrical parameters of the modeled wind farm.

Component	Parameters
Wind farm	6 wind turbines
	1.5 MW, 50 Hz
Transformer 1	575 V/25 kV, 50 Hz
Transformer 2	25 kV/120 kV, 50 Hz

The DFIG is modeled using a vector control strategy in the synchronous reference frame, where the rotor-side converter (RSC) controls the rotor currents to independently regulate the stator active and reactive power, while the grid-side converter (GSC) maintains the DC-link voltage and controls reactive power exchange with the grid. The detailed electrical and control parameters of the wind farm are provided in Table 2^1–3,7^. A 25 kV collector feeder and step-up transformers, which add more impedance between the wind farm and the main transmission line, are used to link the DFIG-based wind farm to the grid. This limits the direct effect of transmission line faults on the dynamics of the RSC. So, detailed modeling of converter protection mechanisms such as the crowbar circuit is ignored because the main goal of this study is transmission line fault classification based on terminal measurements. Therefore, during the simulated fault scenarios it is assumed that the DFIG operates under normal control conditions (Table 3).

**Table 3:** Relationship between the firing angle $\alpha$, inductive reactance ${X}_{LT}(\alpha )$, and the equivalent TCSC reactance ${X}_{TCSC}$ used in the proposed model.

α degree	${X}_{LT}$(α) ohm	${X}_{TCSC}$ ohm	Region
1	2.42	3.02	Inductive
10	2.86	3.68	Inductive
20	3.56	4.59	Inductive
30	4.69	6.47	Inductive
40	6.79	12.72	Inductive
50.77	12.58	Inf	Resonance
60	41.32	− 21.65	Capacitive
70	− 30.99	− 14.11	Capacitive
80	− 11.77	− 12.70	Capacitive
85	− 9.1	− 12.56	Capacitive

Referring to Fig. 1, the TCSC is employed to enhance system stability by dynamically regulating the effective line reactance. The primary function of the TCSC is to compensate a portion of the capacitive reactance ${X}_{CT}$ through controlled variation of the inductive reactance ${X}_{LT}$. This is achieved by adjusting the thyristor firing angle $\alpha$, which determines the equivalent inductive reactance of the TCSC^1,25^. The modified value of the inductive reactance ${X}_{LT}$ as a function of the firing angle is given by the following equation

1 $${X}_{LT}(\alpha )={X}_{LT}\frac{\pi }{\pi -2\alpha -sin\alpha }$$

The firing angle $\alpha$ is measured from the zero crossings of the line current, where ${X}_{LT}$ denotes the TCSC inductive reactance. The effective inductive reactance ${X}_{LT}(\alpha )$ reaches its minimum value when $\alpha ={0}^{\circ }$; in this condition, the majority of the line current flows through the TCSC inductor. This operating state, known as the bypassed mode, corresponds to full thyristor conduction with the valve closed. Conversely, when $\alpha ={90}^{\circ },{X}_{LT}\left(\alpha \right)$ attains its maximum value, resulting in negligible current flow through the TCSC inductive branch. This operating condition is referred to as the blocked mode, in which the thyristor valve is open. For intermediate firing angles ${0}^{\circ }<\alpha <{90}^{\circ }$, the current is shared between the TCSC capacitive reactance ${X}_{CT}$ and inductive reactance ${X}_{LT}$. This partially conducting state is known as the vernier mode. For sub-synchronous resonance (SSR) damping applications, the TCSC is typically operated in the capacitive region^1^.

**Fig. 2:**
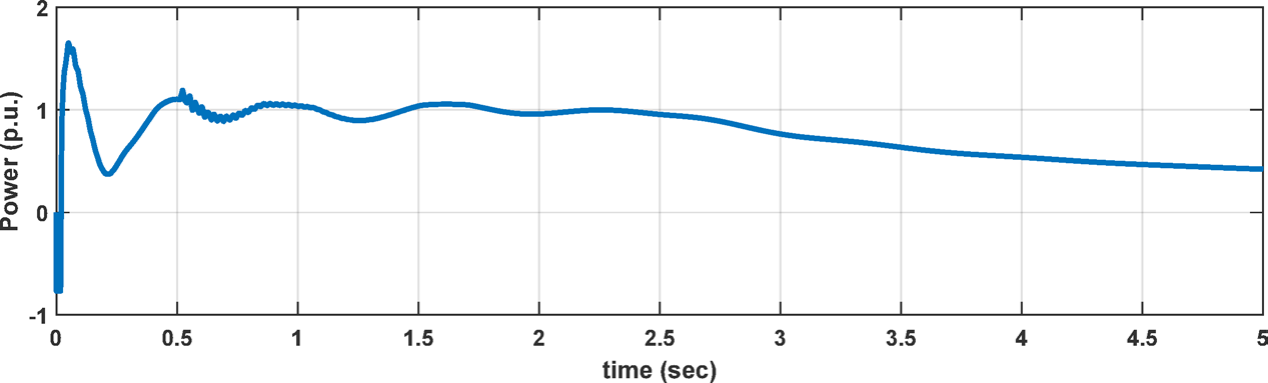
The system’s power dynamic response at 70% compensation level and wind speed change from 15 m/sec to 8 m/sec at 2.5 s.

**Fig. 3:**
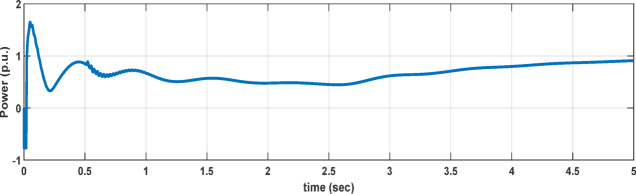
The system’s dynamic power response at 70% compensation level and wind speed changes from 8 m/sec to 15 m/sec at 2.5 s.

**Fig. 4:**
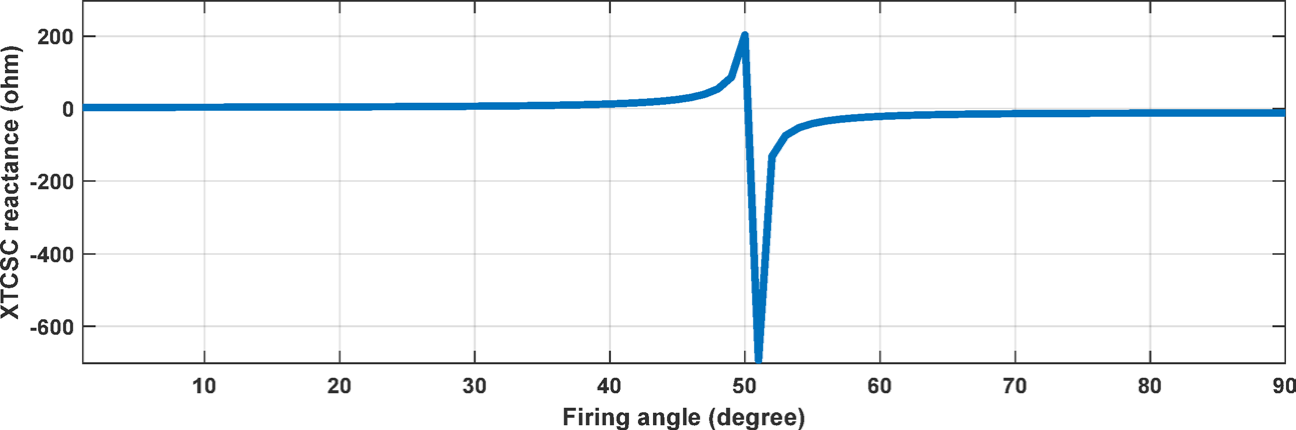
TCSC impedance characteristics when ${\mathrm{X}}_{\mathrm{L}\mathrm{T}}$ = 0.19 ${\mathrm{X}}_{\mathrm{C}\mathrm{T}}.$

In this study, to maintain system stability and suppress oscillations arising from high compensation levels (up to 70%) and time-varying wind speed, a TCSC constant-power control scheme based on a PI controller is employed. The PI-controlled TCSC effectively damps SSR^26^ across different compensation levels, exhibiting faster oscillation convergence, as reported in^1^. As illustrated in Fig. 2, the system remains stable when the wind speed decreases from 15 m/s to 8 m/s, and similarly when the wind speed increases from 8 m/s to 15 m/s, as shown in Fig. 3, while maintaining a compensation level of K = 70%. These results confirm the effectiveness of the PI-controlled TCSC in tracking wind-speed variations and ensuring system stability under high compensation conditions. All TCSC equations used in the model are adopted from^1^. It is significant to highlight that the dynamic variation in TCSC equivalent reactance has a direct impact on the apparent impedance sensed by protection relays during fault conditions, introducing additional challenges to traditional transmission line protection schemes^2,27^. Accordingly, the time-domain simulation results and the TCSC impedance characteristics shown in Fig. 4 confirm that the optimal ratio for maintaining system stability in the studied model is ${X}_{LT}=0.19\hspace{0.17em}{X}_{CT}$.

## Proposed intelligent methodology for TCSC-compensated transmission systems with wind power integration

**Fig. 5:**
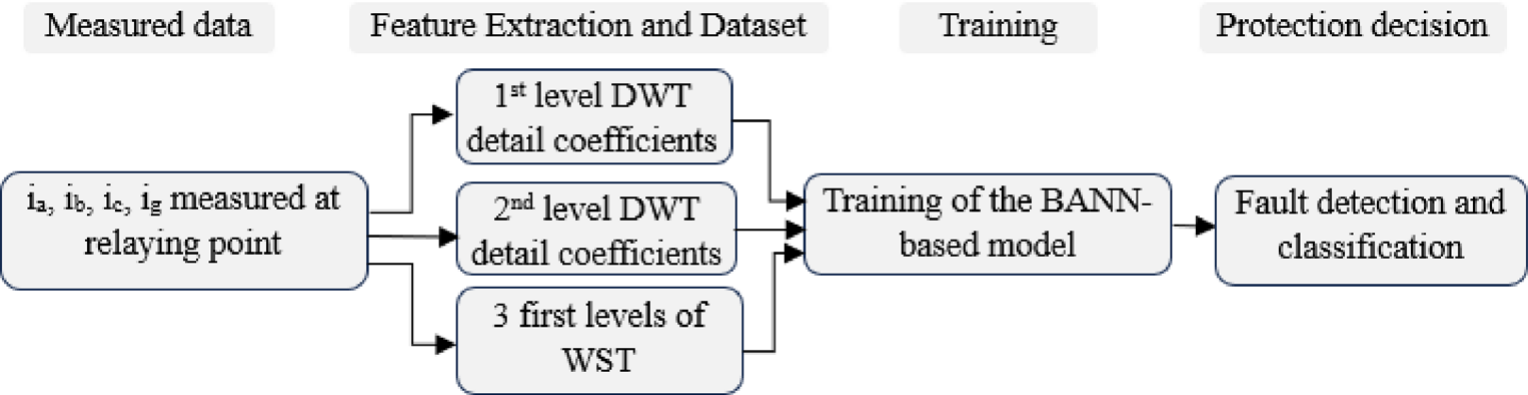
Sequential stages of signal processing, feature extraction, and fault classification.

In this section our suggested protection scheme for fault detection and classification in TCSC-compensated transmission lines integrated with wind power is discussed. The system processes locally measured three-phase current signals and the ground current using combined signal processing techniques to construct three distinct datasets as shown in Figure 5.

Specifically, DWT is utilized to extract detailed coefficients (cD1) and (cD2), while WST analyzes the first three levels to extract spectral energy features. The maximum values of these extracted features constitute input data sets, which are trained and tested using a BANN to achieve precise fault classification and identification.

### Datasets generation via DWT coefficients

**Fig. 6:**
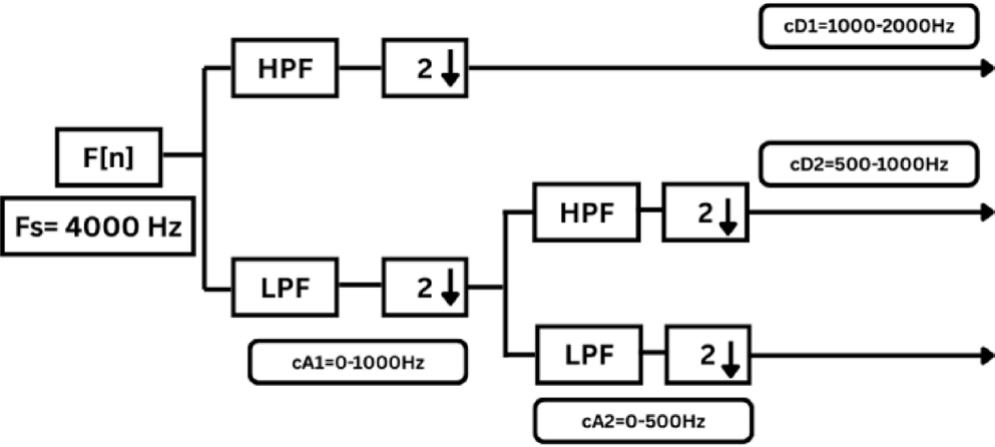
Implementation of DWT using multistage filter bank.

Fault analysis studies are conducted under different operating conditions, including variations in fault location, wind speed, TCSC compensation level, fault inception angle, and fault resistance. For each fault case, the measured three-phase and ground current signals are sampled at 4 kHz and decomposed using the db4 mother wavelet. The first-level detail coefficients (cD1), corresponding to the high-frequency band of 2000–1000 Hz, are extracted to capture transient fault components, as illustrated in Figure 6. The maximum values of these coefficients are used to construct the first feature dataset. The maximum feature value is computed as:

2 $${C}_{D1max}=\underset{1\le i\le N}{\mathrm{max}}({c}_{i})$$

where:

${c}_{i}$ represents the ${i}^{\mathrm{th}}$ DWT coefficient of cD1,

$N$ is the total number of coefficients in the decomposition level cD1.

This maximum value is selected as a representative feature to capture the peak transient energy during fault conditions.

**Fig. 7:**
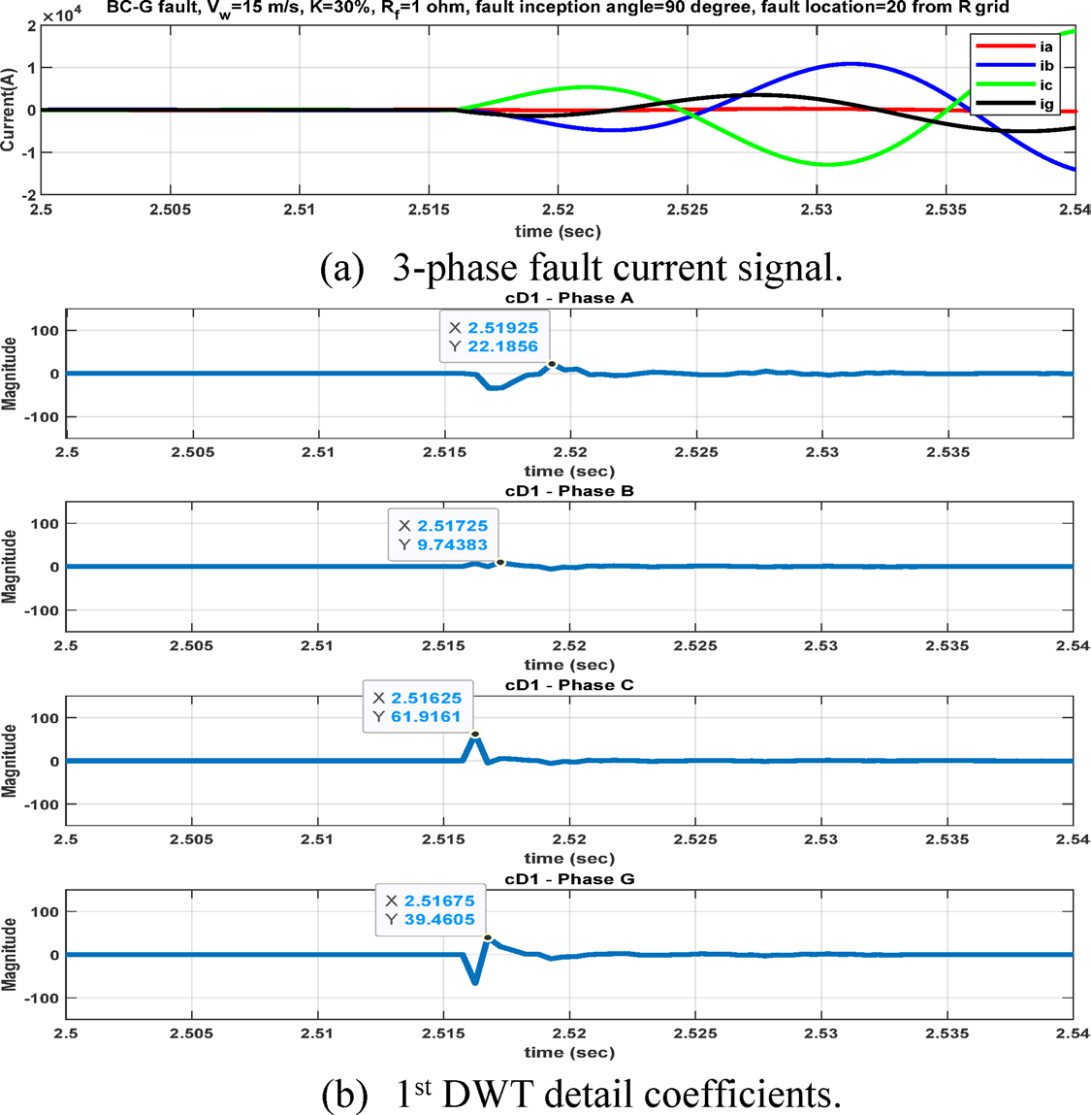
Output of DWT for BC-G fault using cD1 for 15 m/sec wind speed, 30% TCSC compensation level, 1Ω fault resistance, ${90}^{^\circ }$ fault inception angle at 20 km from the grid side.

**Fig. 8:**
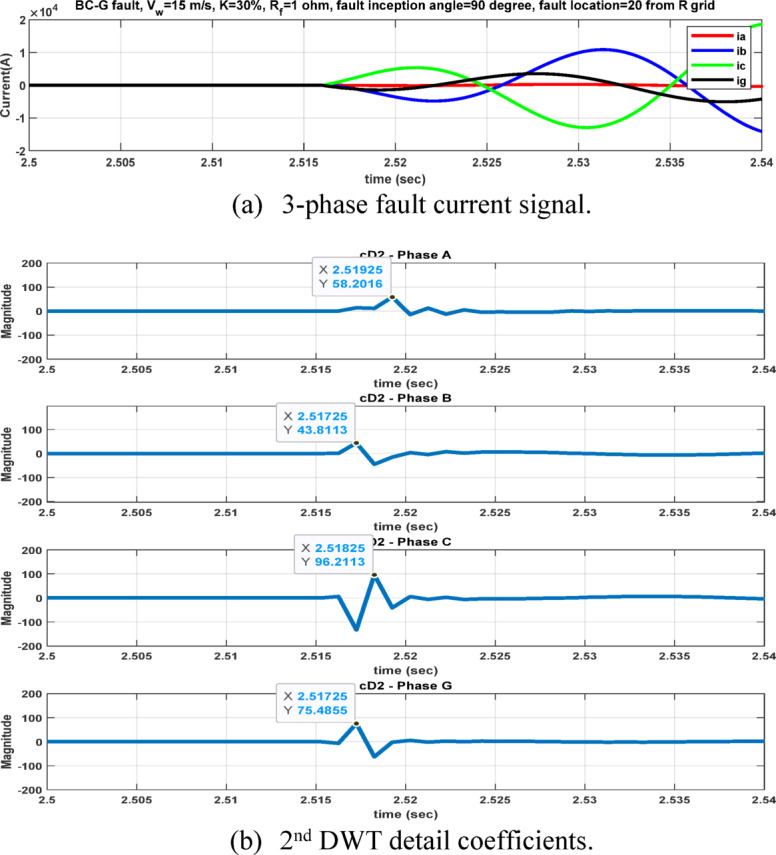
Output of DWT for BC-G fault using cD2 for 15 m/sec wind speed, 30% TCSC compensation level, 1Ω fault resistance, ${90}^{^\circ }$ fault inception angle at 20 km from the grid side.

The second feature dataset is formed using the second-level DWT detail coefficients (cD2), which represent the 1000–500 Hz frequency band as in Fig. 6. The detection and classification performance of the high-frequency (cD1) and lower-frequency (cD2) DWT features are comparatively evaluated to identify the more effective representation. Although the simple implementation and mathematical burden of DWT, its coefficients are strongly affected by the fault inception angle^28^, and then affect the classification accuracy. After extracting the fault coefficients using cD1and cD2 coefficients, it is observed that for a BC–G fault occurring at a fault inception angle of ${90}^{^\circ }$, the resulting maximum coefficient values deviate from the expected fault signature. Specifically, when using both cD1 and cD2, the maximum coefficient of phase-A current exceeds that of phase-B current, despite phase-B being directly involved in the fault. In contrast, when the fault inception angle is changed to ${0}^{^\circ }$ or ${90}^{^\circ }$ the extracted maximum coefficients correctly reflect the faulty phases. This behavior indicates a pronounced sensitivity of DWT-based features to the fault inception angle, as illustrated in Figs. 7 and 8. Consequently, the reliability of fault classification using DWT alone may degrade under certain inception angles, motivating the investigation of more robust feature extraction techniques. Following the calculation of the maximum values of the DWT coefficients for 3240 simulated fault occurrences, the resultant features are structured into a training dataset and provided for the neural network training phase, as seen in Table 4. These outcomes are therefore further compared to a third feature dataset that is derived from the WST coefficients. This dataset produces feature vectors that are invariant to temporal shifts, robust to noise, stable against time-warping deformations, and exhibit low sensitivity to the inception angle variation. It has also recently shown promising results in classification tasks across several applications to evaluate overall robustness and accuracy.

**Table 4:** Sample of the dataset containing maximum DWT coefficients of the current signals for ANN training.

Cases	Wind speed (m/s)	K%	Rf (ohm)	Fault inception angle (Deg)	Fault location (Km)	Fault type	Max(cD1) for phase A current	Max(cD1) for phase B current	Max(cD1) for phase C current	Max(cD1) for ground current	Max(cD2) for phase A current	Max(cD2) for phase B current	Max(cD2) for phase C current	Max(cD2) for ground current
1	15	30	1	0	20	ABC	2.56E + 00	1.12E + 02	1.82E + 01	3.82E-11	1.02E + 02	1.89E + 02	1.31E + 02	6.77E-11
						ABG	1.12E + 01	9.66E + 01	1.56E + 01	2.88E + 01	1.40E + 02	1.51E + 02	4.15E + 01	4.61E + 01
						ACG	6.12E + 00	3.49E + 01	1.01E + 01	6.74E + 01	3.50E + 01	1.51E + 01	6.40E + 01	7.05E + 01
						BCG	9.29E + 00	1.07E + 02	1.96E + 01	1.08E + 01	2.45E + 01	1.63E + 02	1.60E + 02	3.86E + 01
						AB	1.17E + 01	7.19E + 01	6.48E-01	1.96E-02	1.68E + 02	1.36E + 02	3.54E + 00	1.78E-02
						AC	2.44E + 01	1.04E + 00	7.82E + 00	1.83E-02	1.32E + 01	6.39E + 00	2.74E + 01	3.56E-02
						BC	2.20E + 00	9.63E + 01	1.95E + 01	9.15E-03	7.75E + 00	1.50E + 02	1.82E + 02	9.92E-03
						AG	5.18E + 00	1.27E + 01	1.27E + 01	2.27E + 01	6.27E + 01	1.69E + 01	1.64E + 01	4.93E + 01
						BG	2.52E + 01	8.89E + 01	2.47E + 01	7.18E + 01	6.65E + 01	1.07E + 02	6.35E + 01	1.17E + 02
						CG	2.75E + 01	2.77E + 01	9.12E + 00	5.76E + 01	2.93E + 01	2.92E + 01	8.08E + 01	6.08E + 01
5	15	30	1	90	20	ABC	2.25E + 01	2.82E + 01	8.30E + 01	3.91E-11	2.10E + 02	1.53E + 01	1.56E + 02	4.78E-11
						ABG	1.70E + 01	4.34E + 01	2.66E + 01	5.03E + 01	1.55E + 02	6.86E + 01	1.14E + 01	5.85E + 01
						ACG	1.95E + 01	7.46E + 00	8.88E + 01	1.63E + 01	2.05E + 02	9.01E + 00	1.63E + 02	4.51E + 00
						BCG	2.22E + 01	9.74E + 00	6.19E + 01	3.95E + 01	5.82E + 01	4.38E + 01	9.62E + 01	7.55E + 01
						AB	1.58E + 01	6.97E + 01	8.31E-01	1.39E-02	1.14E + 02	9.27E + 01	3.49E + 00	2.67E-02
						AC	1.80E + 01	1.42E + 00	9.71E + 01	4.06E-03	2.02E + 02	5.82E + 00	1.65E + 02	8.31E-03
						BC	9.42E-01	2.27E + 00	2.74E + 01	2.79E-02	2.12E + 00	8.85E + 01	7.07E + 01	2.59E-02
						AG	1.33E + 01	3.91E + 01	3.94E + 01	7.96E + 01	1.29E + 02	4.33E + 01	4.31E + 01	9.83E + 01
						BG	5.28E + 00	2.12E + 01	5.33E + 00	1.86E + 01	1.58E + 01	9.23E + 00	1.61E + 01	1.13E + 01
						CG	1.90E + 01	1.87E + 01	6.64E + 01	5.29E + 01	4.88E + 01	4.74E + 01	8.83E + 01	9.81E + 01
9	15	30	1	135	20	ABC	1.41E + 01	8.30E + 00	1.16E + 02	4.25E-11	5.69E + 01	2.54E + 01	2.96E + 02	6.22E-11
						ABG	6.55E + 00	6.44E + 00	3.56E + 01	6.94E + 01	3.69E + 01	1.57E + 01	4.79E + 01	1.38E + 02
						ACG	1.77E + 01	1.41E + 01	1.05E + 02	2.06E + 01	6.08E + 01	8.39E + 00	2.62E + 02	3.36E + 01
						BCG	9.91E + 00	1.33E + 01	1.05E + 02	2.03E + 01	6.96E + 00	3.04E + 01	2.60E + 02	4.45E + 01
						AB	2.90E + 00	5.60E-01	8.27E-01	1.88E-02	2.11E + 01	1.19E + 01	6.11E + 00	3.40E-02
						AC	1.83E + 01	1.45E + 00	8.59E + 01	1.60E-02	6.31E + 01	6.27E + 00	2.24E + 02	1.85E-02
						BC	6.88E-01	1.53E + 01	8.75E + 01	1.32E-02	1.75E + 00	4.24E + 01	2.21E + 02	1.18E-02
						AG	6.12E + 00	1.87E + 01	1.88E + 01	4.02E + 01	2.49E + 01	2.02E + 01	1.95E + 01	8.99E + 01
						BG	2.24E + 01	8.44E + 00	2.25E + 01	4.25E + 01	3.22E + 01	1.97E + 01	3.19E + 01	8.93E + 01
						CG	2.50E + 01	2.59E + 01	9.14E + 01	7.42E + 01	3.31E + 01	3.69E + 01	1.96E + 02	1.20E + 02

### Datasets generation via WST coefficients

**Fig. 9:**
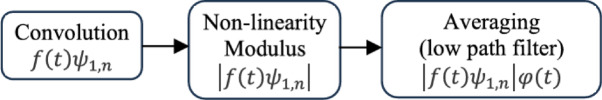
Block diagram illustrating the mathematical stages of the WST.

**Fig. 10:**
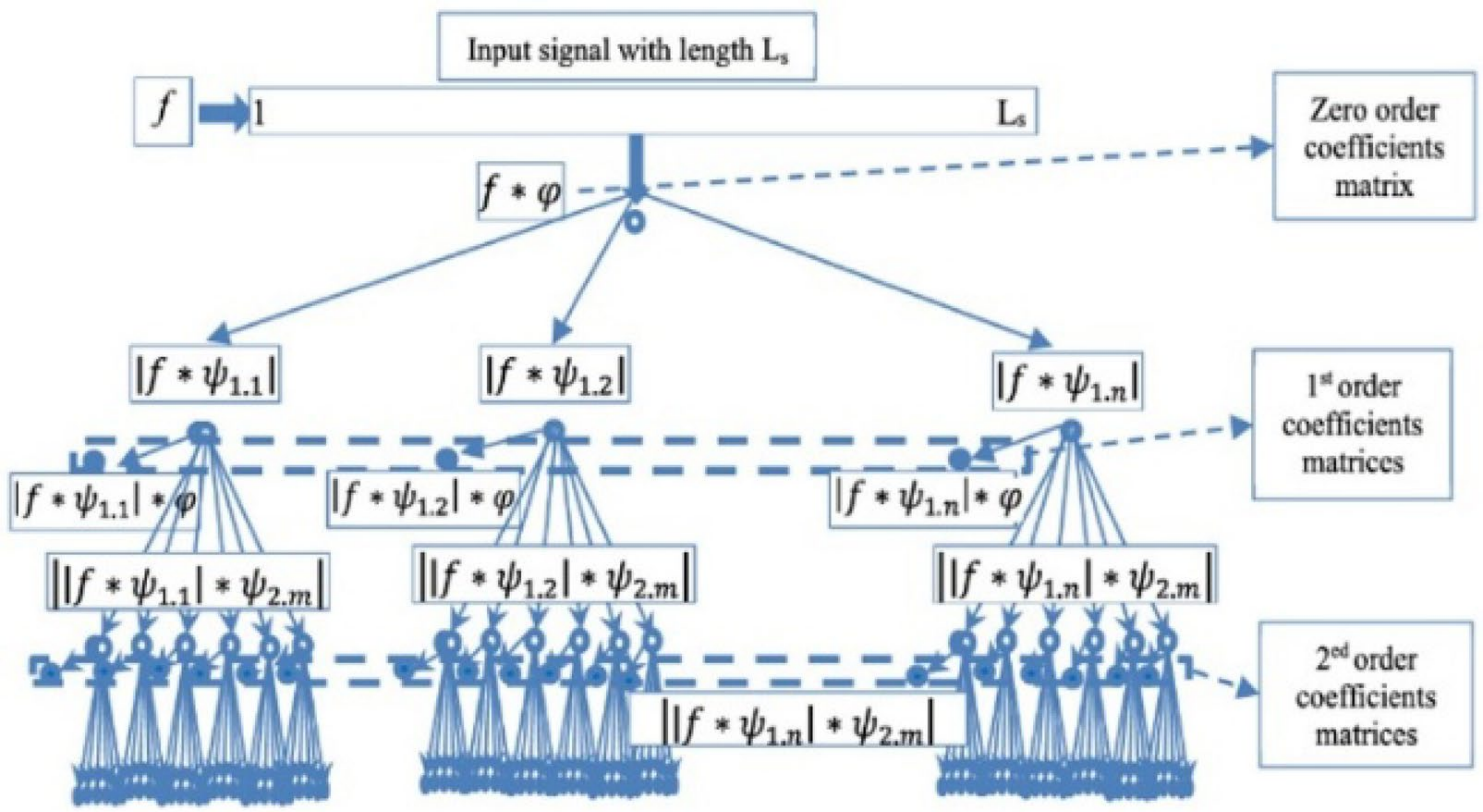
Wavelet time scattering network^21,22^.

In the process of power system disturbance identification and fault classification, the studies reported in^21,22^ demonstrate that the wavelet scattering transform (WST) is capable of extracting discriminative features from current signals, yielding promising classification performance. In the first layer, it can extract the fundamental and nearby frequencies; in the second layer, it can extract the high-frequency components; and the DC components related to the fault initiation can be extracted in the zero layer. More than 98% of the input signal energy is carried by the zero, first-, and second-order coefficients, as demonstrated by Andén and Mallat in^23^. This suggests that the feature matrix containing the characteristics recorded at the first three levels is adequate for classification issues. Bruna and Mallat^24^ introduced the mathematical formulation and hierarchical structure of the WST that combines the advantages of wavelet analysis with the hierarchical architecture of convolutional neural networks (CNNs). As illustrated in Fig. 9, WST analyzes real-valued signals through three successive stages to construct a sequence of scattering coefficients that are invariant to time shifts and less sensitive to fault inception angle through cascaded wavelet convolutions, modulus nonlinearities, and averaging operations. Unlike DWT, the squared magnitudes of the real and imaginary components of the convolved signal are averaged to yield modulated coefficients that are robust to temporal translations of the input signal making it particularly suitable for power system fault classification under highly dynamic conditions. From the time scattering network of WST in Fig. 10, the coefficients at different decomposition layers are computed. The zero-order scattering coefficient represents the averaged value of the input signal obtained through convolution with a low-pass scaling ($\boldsymbol{\varphi }$) function, capturing the DC component of the current signal as in Eq. (3).

3 $${S}_{0}=f(t)\varphi (t)$$

Then the first order scattering coefficients are obtained by computing the modulus of the input signal convolved with a family of wavelets at different scales, followed by averaging through a low-pass scaling function as:

4 $${S}_{1}=\left|f(t){\psi}_{1,n}\right|\varphi (t)$$

where *n* refers to the number of filters in the first order filter bank. The second scattering layer is obtained by further convolving the first-layer modulus coefficients with wavelets and applying the modulus operation, enabling the extraction of higher-order modulation features as:

5 $${S}_{2}=\left|\left|f(t){\psi}_{1,n}\right|{\psi}_{2,m}\right|\varphi (t)$$

where *m* refers to the number of filters in the second filter bank. Information lost at earlier levels is restored by computing the upper levels.

A WS network with 150 paths and 16 coefficients in each path is generated using a sampling rate of 4000 Hz, an invariance scale of 0.32 seconds, and the quality factors of the first- and second-order filter banks are set to Q_1_ = 8 and Q_2_ = 1, and a signal length of 4096 samples. An invariance scale of 0.32 sec means the WS network is stable to time shifts up to 320 ms, making it suitable for analyzing non-stationary signals such as power system disturbances, vibrations, or fault transients.

**Fig. 11:**
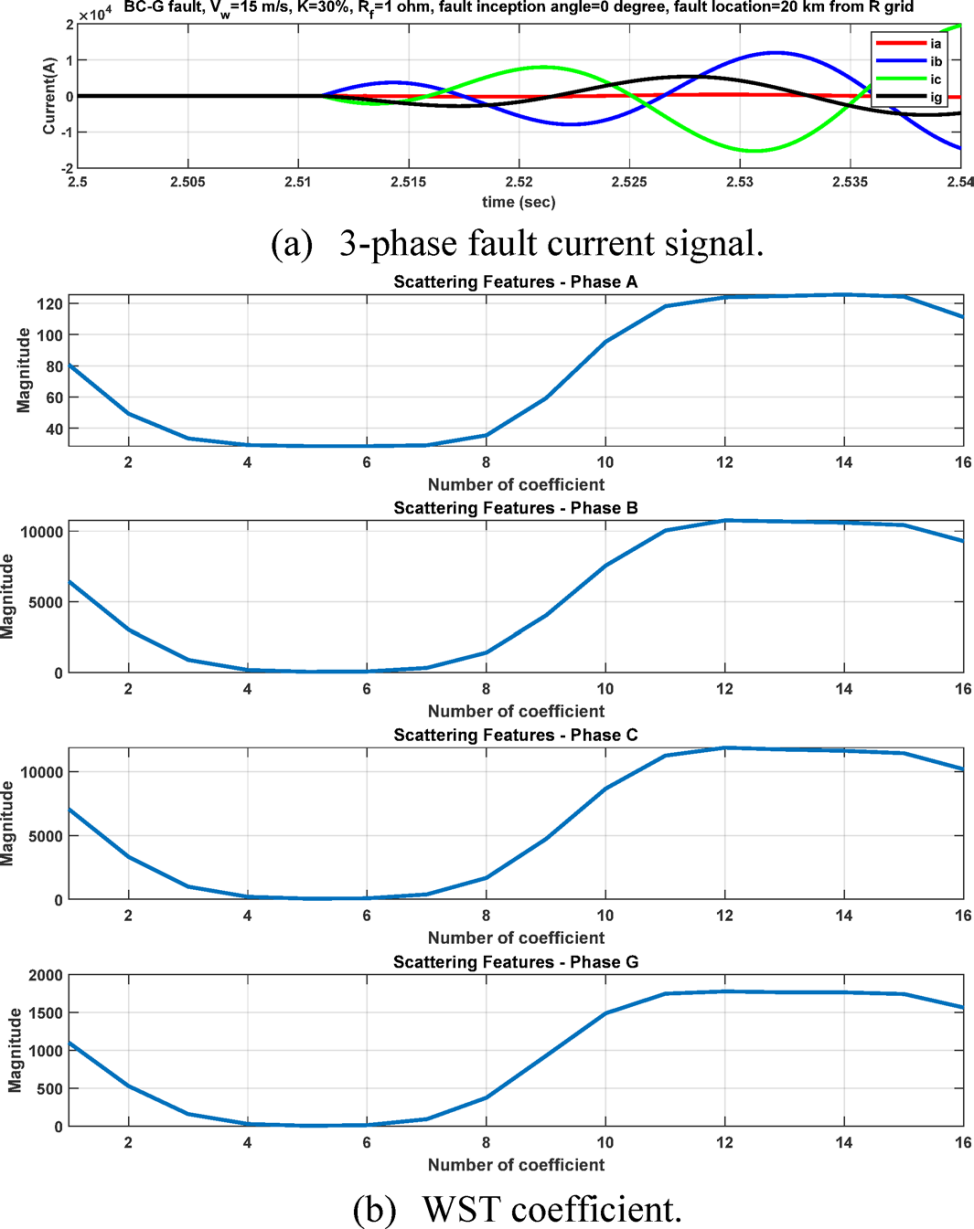
The 44th WST path for BC-G fault using 4096 samples, [8 1] quality factor and 4000 Hz sampling rate at wind speed 15 m/sec, 30% TCSC compensation level, 1 Ω fault resistance, ${0}^{^\circ }$ fault inception angle at 20 km from the grid side.

**Fig. 12:**
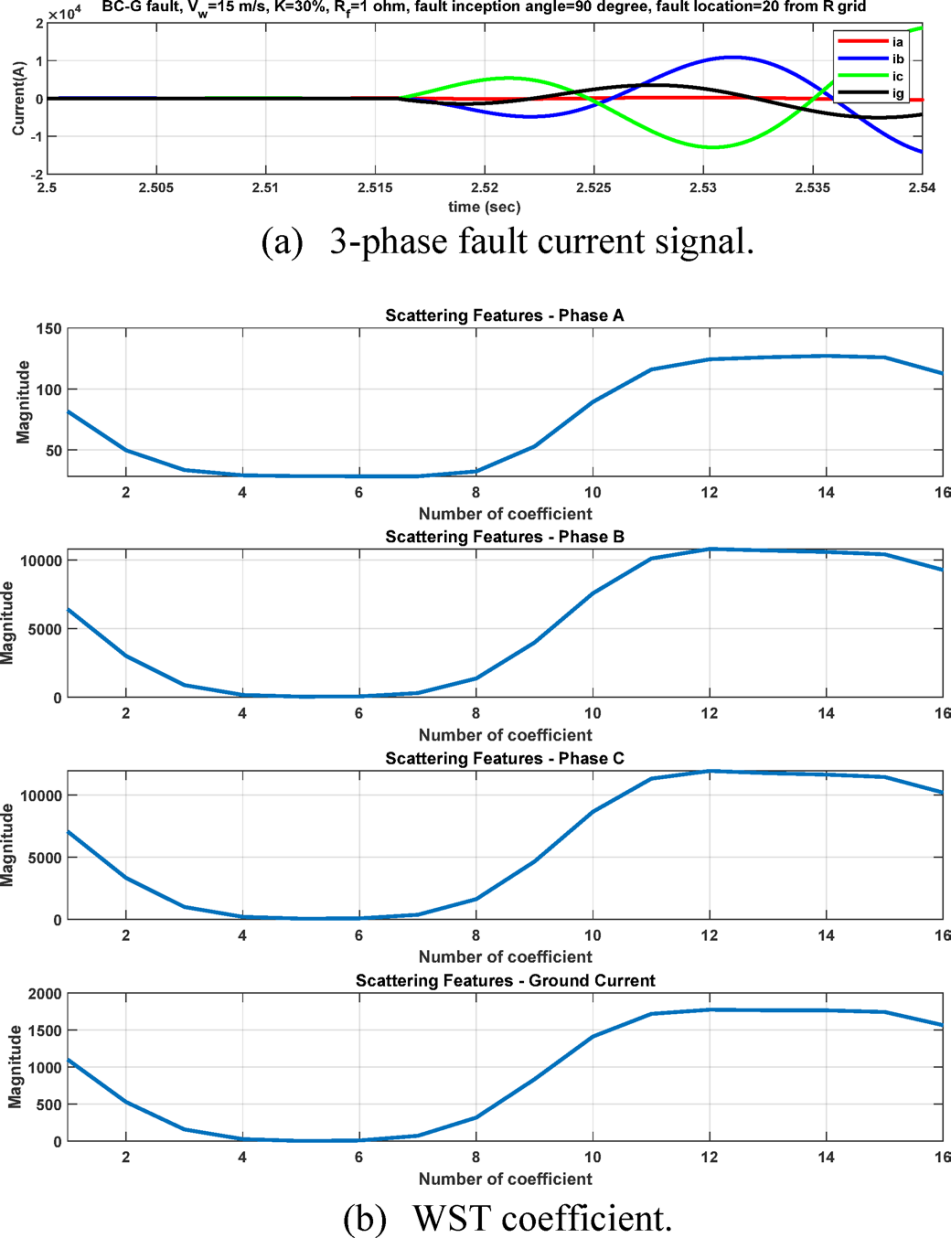
The 44th WST path for BC-G fault using 4096 samples, [8 1] quality factor and 4000 Hz sampling rate for 15 m/sec wind speed, 30% TCSC compensation level, 1 Ω fault resistance, ${90}^{^\circ }$ fault inception angle at 20 km from the grid side.

After extracting the WST features from each current (${\mathrm{i}}_{\mathrm{a}},{\mathrm{i}}_{\mathrm{b}},{\mathrm{i}}_{\mathrm{c}} \mathrm{a}\mathrm{n}\mathrm{d} {\mathrm{i}}_{\mathrm{g}})$, the resulting feature matrix has dimensions of 4 × 150 × 16. In our study, the 44th row consistently contains the maximum value, as it corresponds to the scattering path tuned to the fundamental frequency^21,22^ . After computing the maximum values of the WST coefficients for 3240 simulated fault cases, the resulting features are organized into a training dataset and prepared for the neural network training stage, see Table 5. As illustrated in Figs. 11 and 12, it is important to note that variations in the fault inception angle do not significantly affect the WST output features.

**Table 5:** Sample of the dataset containing maximum WST coefficients of the current signals for ANN training.

S.NO	Wind speed (m/s)	K%	Rf (ohm)	Fault inception angle (Deg)	Fault location (Km)	Fault Type	Max (WST coefficients) for phase A current	Max (WST coefficients) for phase B current	Max (WST coefficients) for phase C current	Max (WST coefficients) for ground current
1	15	30	1	0	20	ABC	1.31E + 04	1.30E + 04	1.31E + 04	1.27E-08
						ABG	1.08E + 04	1.19E + 04	1.26E + 02	1.78E + 03
						ACG	1.19E + 04	1.28E + 02	1.08E + 04	1.77E + 03
						BCG	1.26E + 02	1.08E + 04	1.19E + 04	1.78E + 03
						AB	1.13E + 04	1.13E + 04	3.30E + 01	1.70E-02
						AC	1.14E + 04	3.25E + 01	1.13E + 04	1.70E-02
						BC	3.19E + 01	1.13E + 04	1.13E + 04	1.70E-02
						AG	3.52E + 03	2.20E + 02	1.80E + 02	3.13E + 03
						BG	1.80E + 02	3.51E + 03	2.20E + 02	3.12E + 03
						CG	2.20E + 02	1.80E + 02	3.52E + 03	3.12E + 03
5	15	30	1	90	20	ABC	1.30E + 04	1.31E + 04	1.31E + 04	1.28E-08
						ABG	1.08E + 04	1.19E + 04	1.27E + 02	1.78E + 03
						ACG	1.19E + 04	1.25E + 02	1.08E + 04	1.78E + 03
						BCG	1.27E + 02	1.08E + 04	1.19E + 04	1.77E + 03
						AB	1.13E + 04	1.13E + 04	3.30E + 01	1.70E-02
						AC	1.13E + 04	3.20E + 01	1.13E + 04	1.70E-02
						BC	3.22E + 01	1.13E + 04	1.14E + 04	1.70E-02
						AG	3.51E + 03	2.20E + 02	1.80E + 02	3.12E + 03
						BG	1.81E + 02	3.53E + 03	2.21E + 02	3.13E + 03
						CG	2.20E + 02	1.80E + 02	3.52E + 03	3.12E + 03
9	15	30	1	135	20	ABC	1.31E + 04	1.31E + 04	1.30E + 04	1.27E-08
						ABG	1.08E + 04	1.19E + 04	1.28E + 02	1.77E + 03
						ACG	1.19E + 04	1.26E + 02	1.08E + 04	1.78E + 03
						BCG	1.25E + 02	1.08E + 04	1.19E + 04	1.78E + 03
						AB	1.13E + 04	1.14E + 04	3.33E + 01	1.70E-02
						AC	1.13E + 04	3.21E + 01	1.13E + 04	1.70E-02
						BC	3.19E + 01	1.13E + 04	1.13E + 04	1.70E-02
						AG	3.53E + 03	2.20E + 02	1.81E + 02	3.13E + 03
						BG	1.81E + 02	3.52E + 03	2.20E + 02	3.13E + 03
						CG	2.20E + 02	1.80E + 02	3.51E + 03	3.12E + 03

### ANN-based training methodology

**Fig. 13:**
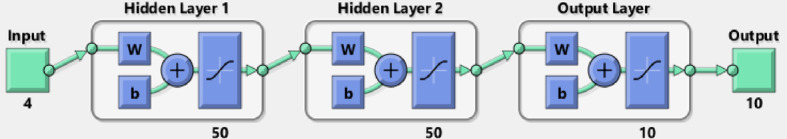
ANN Architecture for fault classification.

**Fig. 14:**
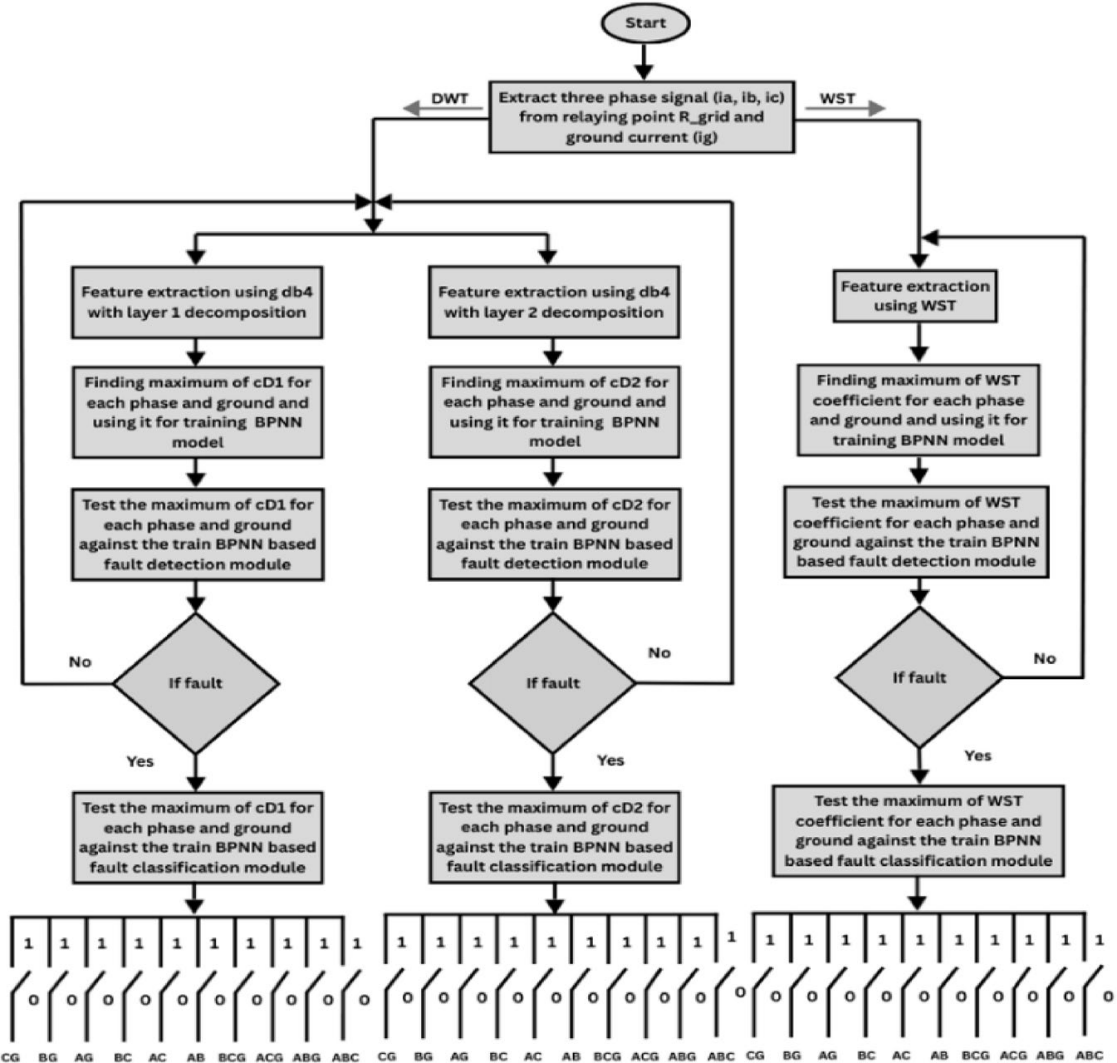
The flowchart of the proposed algorithm.

**Fig. 15:**
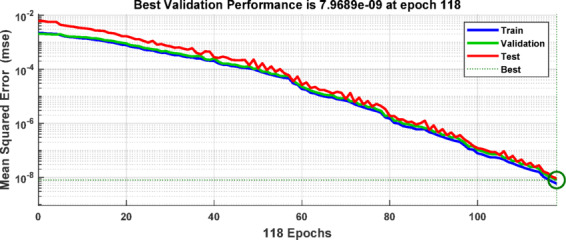
Validation performance of the ANN-based fault detection using Max(cD1) of DWT as the input feature matrix.

**Fig. 16:**
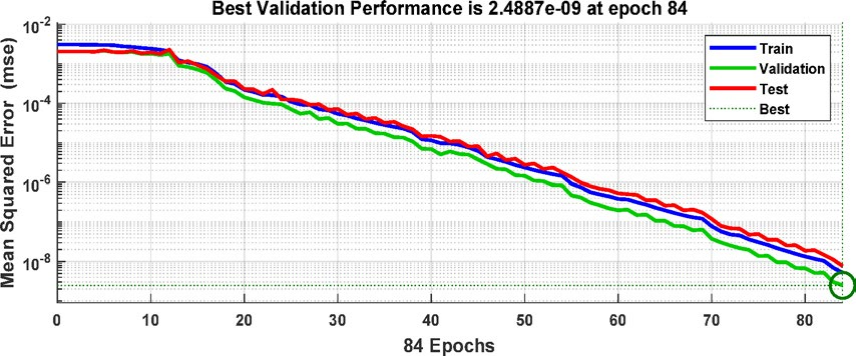
Validation performance of the ANN-based fault detection using Max(cD2) of DWT as the input feature matrix.

**Fig. 17:**
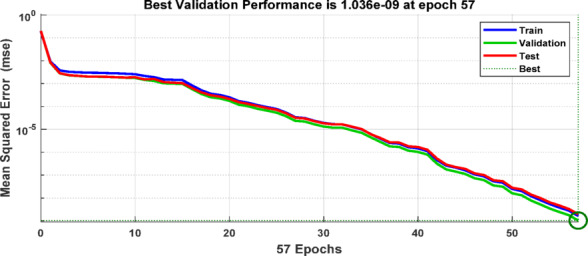
Validation performance of the ANN-based fault detection using Max (WST coefficients) as the input feature matrix.

During the training and testing phases, the BPNN uses the training data sets that were created by extracting the discriminative features from the ground current and three-phase current signals measured at the grid-side relaying point. Based on the extracted input features corresponding to various fault types, as summarized in Table 6, the target outputs are defined using binary labels, where “0” denotes the normal operating condition and “1” represents the faulty condition. The multi-layer feed-forward ANN is trained using the Levenberg Marquardt back-propagation algorithm implemented in MATLAB. Figure 13 depicts the implemented ANN architecture, with an input layer including four neurons (corresponding to the three-phase and ground current signals), two hidden layers each comprising 50 neurons, and an output layer with 10 neurons (represent the 10 shunt fault types). The resulting training dataset forms a 4×3240 feature matrix. The tangent sigmoid activation function is employed in both the hidden layers and the output layer. The training performance is evaluated using the mean squared error (MSE) criterion. Figure 14 presents the flowchart of the proposed algorithm. Figure 15 indicates the MSE convergence when the maximum first-level DWT detail coefficients are used as input features, Figure 16 shows the corresponding results for the maximum second-level DWT detail coefficients, and Figure 17 illustrates the training performance obtained using the maximum WST coefficients.

**Table 6:** Binary encoding of fault classification for various fault types.

Type of Fault	ABC	ABG	ACG	BCG	AB	AC	BC	AG	BG	CG
ABC	1	0	0	0	0	0	0	0	0	0
AB–G	0	1	0	0	0	0	0	0	0	0
AC–G	0	0	1	0	0	0	0	0	0	0
BC–G	0	0	0	1	0	0	0	0	0	0
A–B	0	0	0	0	1	0	0	0	0	0
A–C	0	0	0	0	0	1	0	0	0	0
B–C	0	0	0	0	0	0	1	0	0	0
A–G	0	0	0	0	0	0	0	1	0	0
B–G	0	0	0	0	0	0	0	0	1	0
C–G	0	0	0	0	0	0	0	0	0	1

## Simulation results and discussion

For a total of 3240 simulated fault cases generated using the network model shown in Figure 1 under the operating conditions listed in Table 7; four current signals are captured and processed using DWT and WST to construct the feature datasets. Three distinct datasets are then trained offline using the ANN architecture described in the previous section. Subsequently, an independent set of 480 fault cases, summarized in Table 8 is employed to test and evaluate the detection and classification accuracy and generalization performance of the proposed algorithm.

**Table 7:** Parameters and operating conditions for the 3240 simulated training fault cases.

Parameters	Training
Fault type	AG, BG, CG, AB, AC, BC, ABG, ACG, BCG, BC
Fault location from grid side (Km)	20, 40,60, 80
Fault Inception angle (Deg)	0, 90, 135
Fault Resistance (ohm)	1, 50,100
Compensation level (K%)	30, 50, 70
Wind Speed (m/s)	9, 12, 15
Total number of fault cases generated	10 × 4 × 3 × 3 × 3 × 3 = 3240

**Table 8:** Parameters and operating conditions for the 480 simulated testing fault cases.

Parameters	Testing
Fault types	AG, BG, CG, AB, AC, BC, ABG, ACG, BCG, BC
Fault location from grid side (Km)	30, 50, 70
Fault Inception angle (Deg)	60, 120
Fault Resistance (ohm)	20, 120
Compensation level (K%)	20, 60
Wind Speed (m/s)	8,16
Total number of fault cases generated	10 × 3 × 2 × 2 × 2 × 2 = 480
Dynamic load switching	60% load switched at 2.511 s for compensation level = 70% and wind speed = 8 m/sec. also, for compensation level 20%, 60% and wind speed 8, 12, 16 m/sec
Capacitor switching	0.88 µF capacitor per phase switched on at 2.511 s and switched off at 2.52 s during compensation level = 70% and wind speed = 8 m/s
CT saturation	ABC fault at 2.5185 s causing CT saturation for compensation level = 70%, wind speed = 8 m/sec, 0.1Ω fault resistance and fault location = 85 km from the grid side
Noise in the current signal	with SNR = 10 dB and 30 dB

**Fig. 18:**
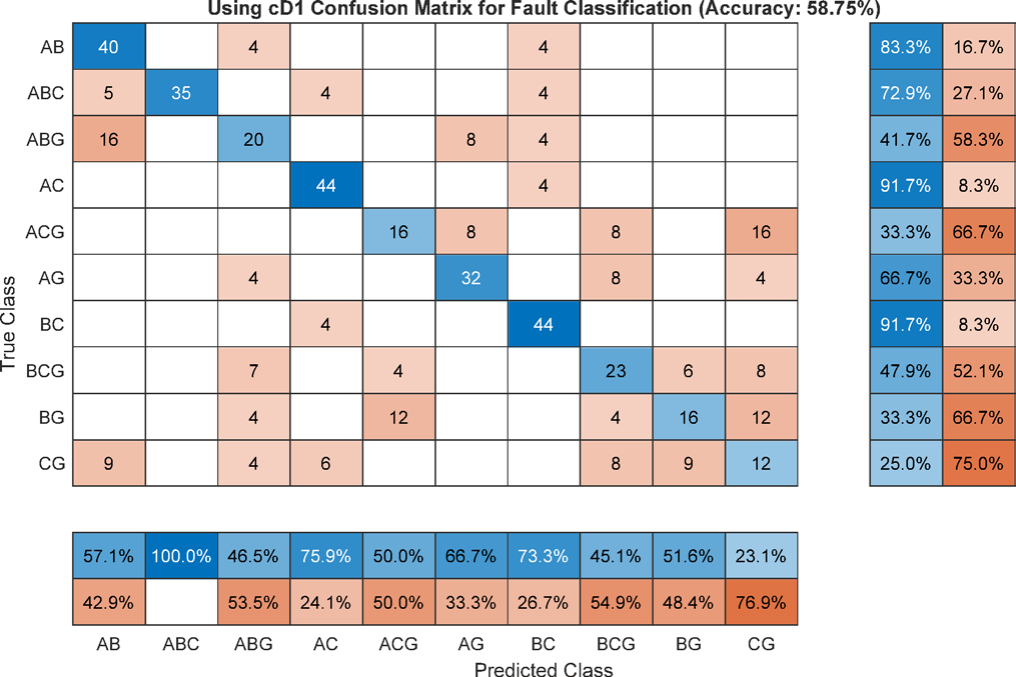
Confusion matrix using max(cD1) of DWT as classifier for fault classification.

**Fig. 19:**
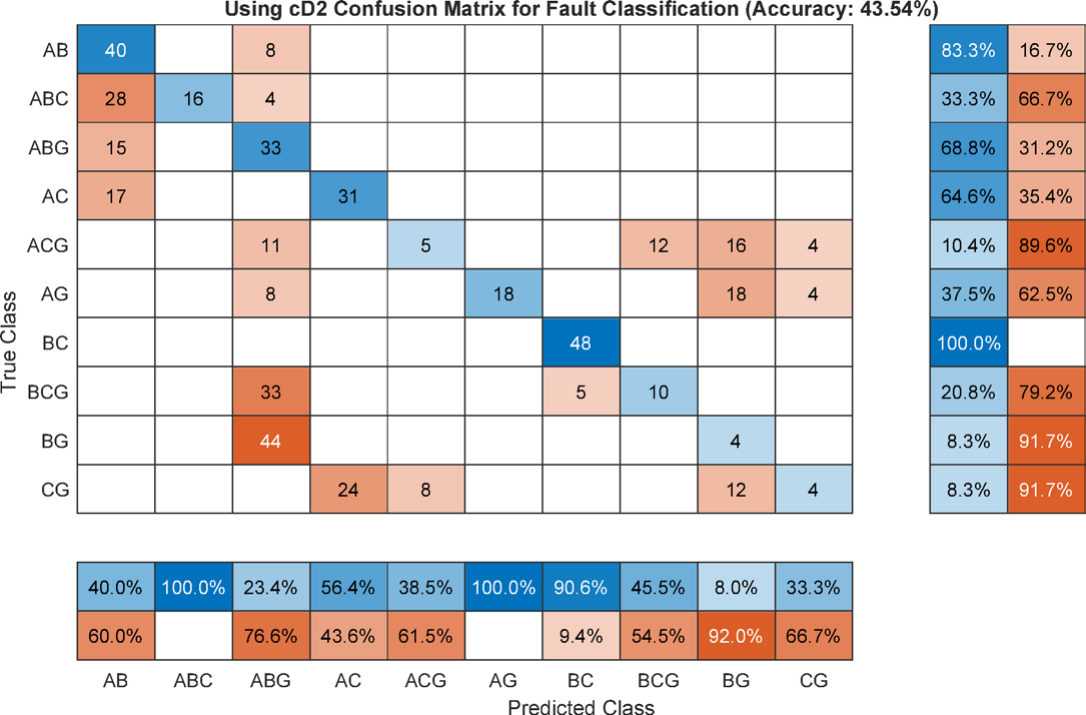
Confusion matrix using max(cD2) of DWT as classifier for fault classification.

**Fig. 20:**
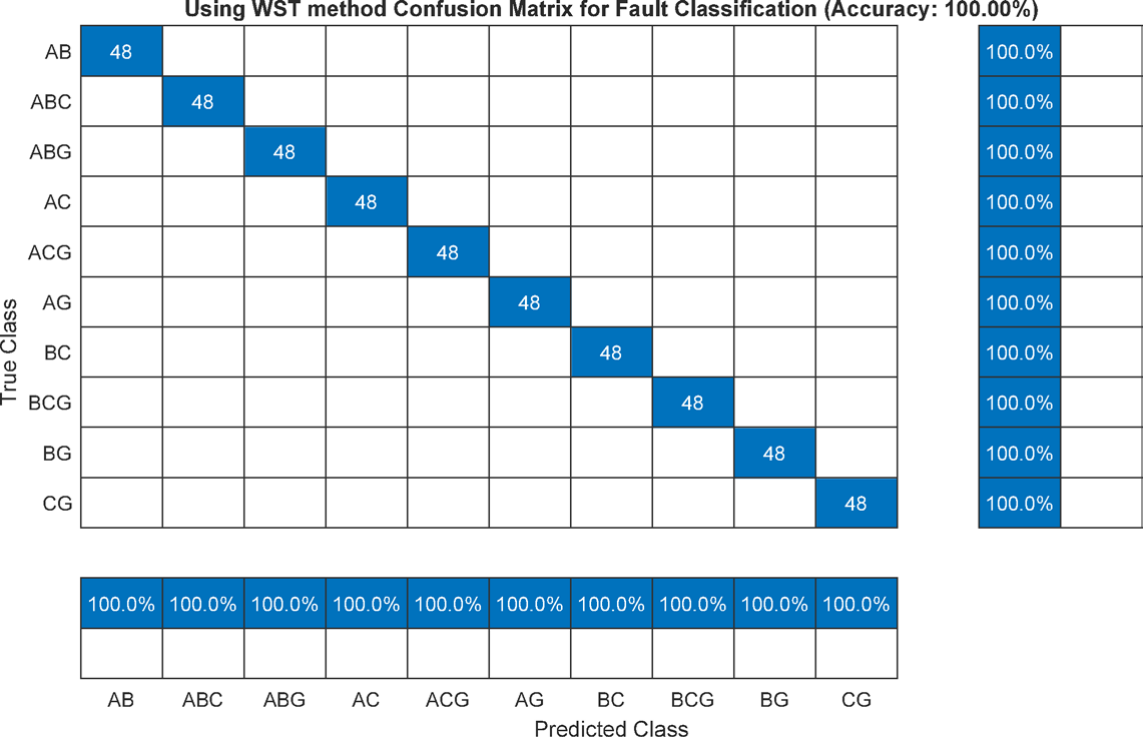
Confusion matrix using max feature of WST as classifier for fault classification.

The overall confusion matrix indicates that the fault classification accuracy achieved using the maximum value of the cD1 is 58.75% as illustrated in Figure 18. When the maximum value of the second-level DWT detail coefficient, cD2 is employed as the classification feature the accuracy decreases to 43.54%, as shown in Figure 19. In contrast, utilizing the maximum feature extracted from the WST yields a classification accuracy of 100% as indicated in Figure 20. It is noted that the classification performance of the DWT-based feature matrix varies significantly with the selected decomposition level. Moreover, the optimal decomposition level is strongly dependent on the sampling rate; consequently, any change in the sampling frequency leads to a substantial variation in the extracted features and hence the classification results. This sensitivity to both decomposition level and sampling rate represents a serious limitation of DWT-based applications for fault classification beside its sensitivity to inception angle.

**Fig. 21:**
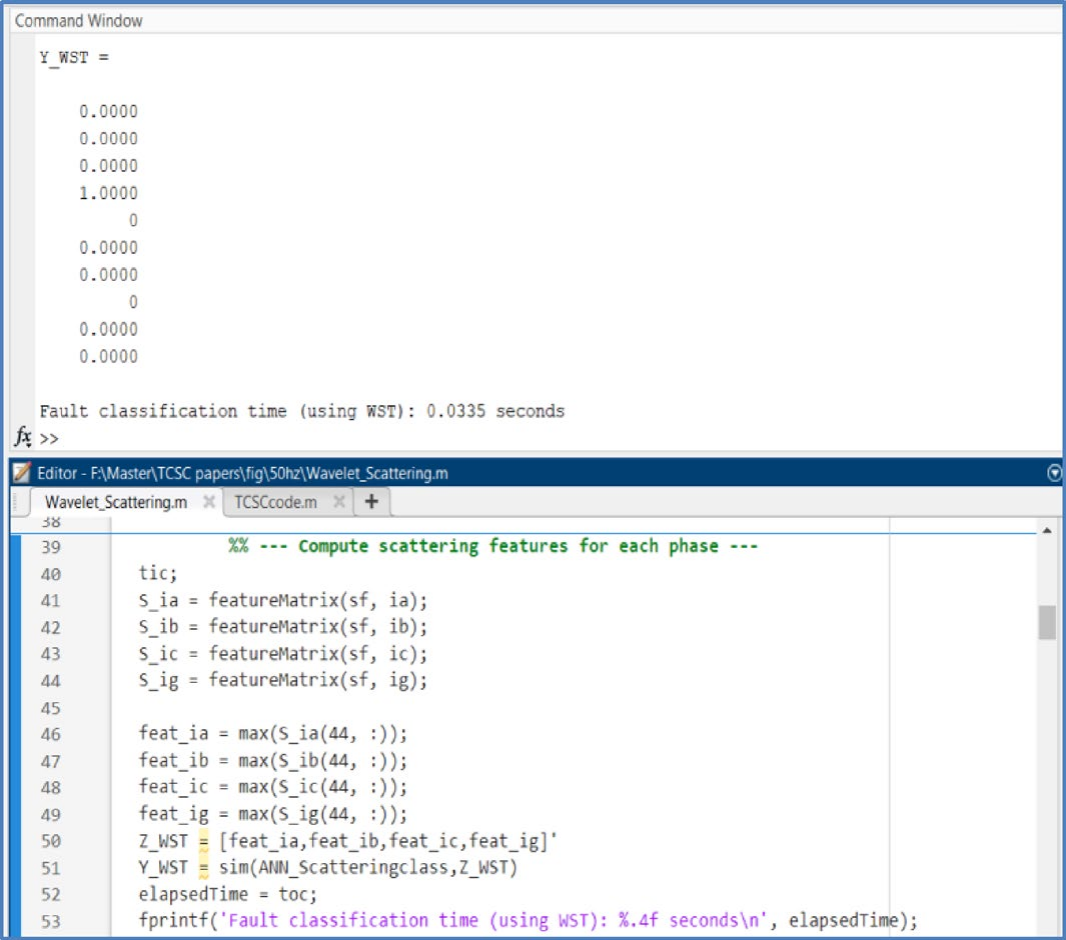
The suggested algorithm’s execution time was measured using MATLAB code.

A computer system with an Intel® Core™ i7-13700H processor running at a base frequency of 2.40 GHz was used to obtain all simulation findings for this investigation. The system was running a 64-bit version of Windows 11 and had 16 GB of RAM installed. MATLAB R2024a was used to perform all simulation tasks, while the training process of the ANN was carried out using MATLAB R2018a. The total computational time required to analyze the measured current signal using the WST and to perform fault classification using the trained BPNN was approximately 0.0335 sec, as illustrated in Figure 21. However, for real-time implementation, the suggested approach does not always require the entire 1.024 second window to make a decision. In reality, the approach can be implemented using a moving (sliding) window approach, in which the feature set is continuously updated as new samples become available. This enables the protection decision to be made well before the entire window is completed with the faulted samples, considerably shortening the effective operating period. Although its computational time is slightly larger than that of the DWT decomposition level 1&2, a comparative evaluation of the three-feature matrix performance that is summarized in Table 9 indicates that the WST-based approach demonstrates superior performance in terms of robustness, classification accuracy, and computational efficiency.

**Table 9:** Comparison between WST-based and DWT-based approaches.

Parameter	Method used		
	DWT (cD1)	DWT (cD2)	WST
FD accuracy in%	97.55	98.57	100
FC accuracy in%	58.75	43.54	100
FD time in ms	5–10	5–10	32–36
FC time in ms	5–10	5–10	32–36
Affected by dynamic load switching?	Yes	Yes	No
Affected by capacitor switching?	Yes	Yes	No

**Fig. 22:**
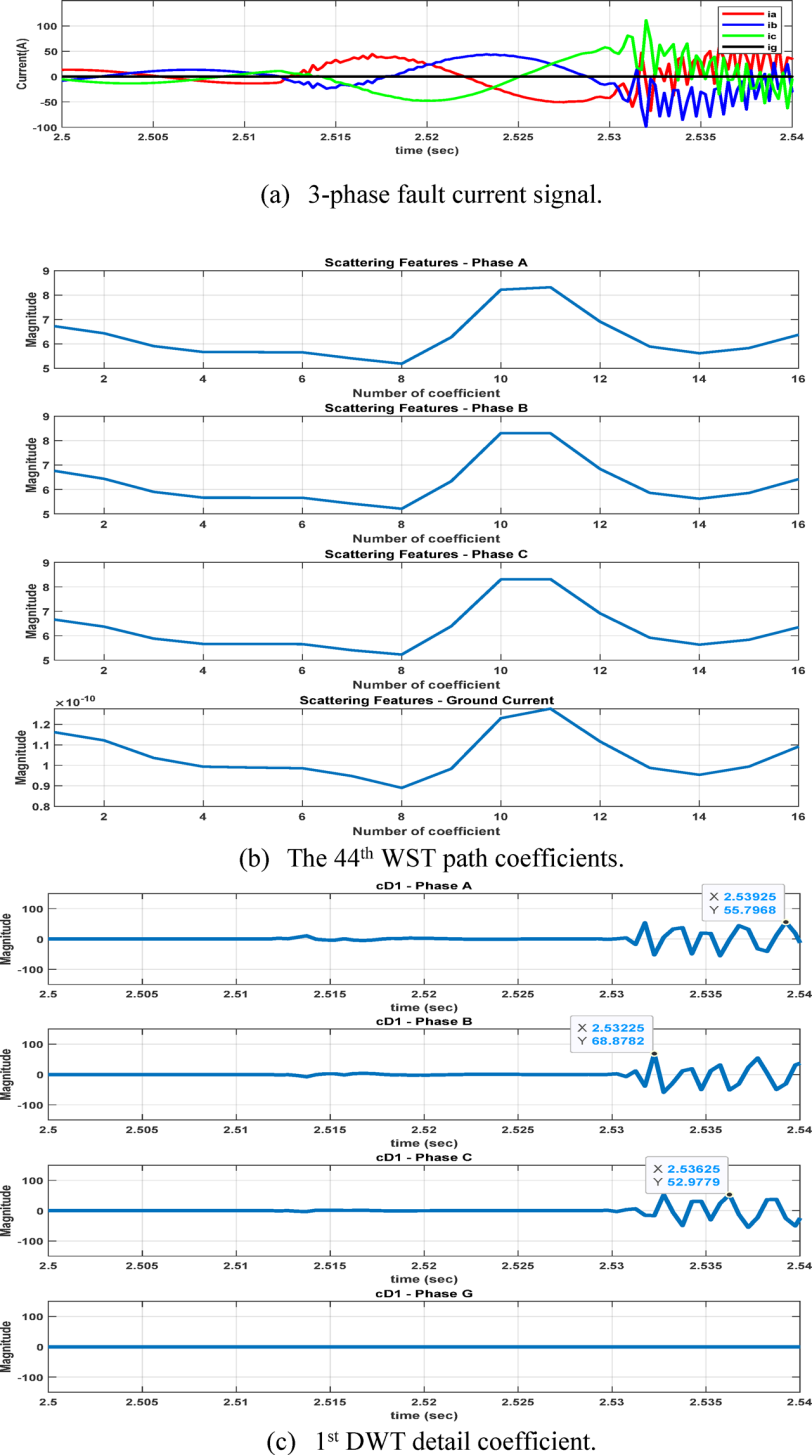

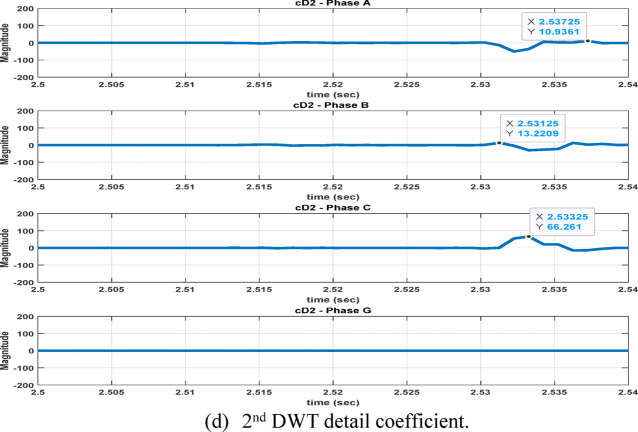
The four measured signals, their WST and DWT coefficients during load switching on at 2.511s.

**Fig. 23:**
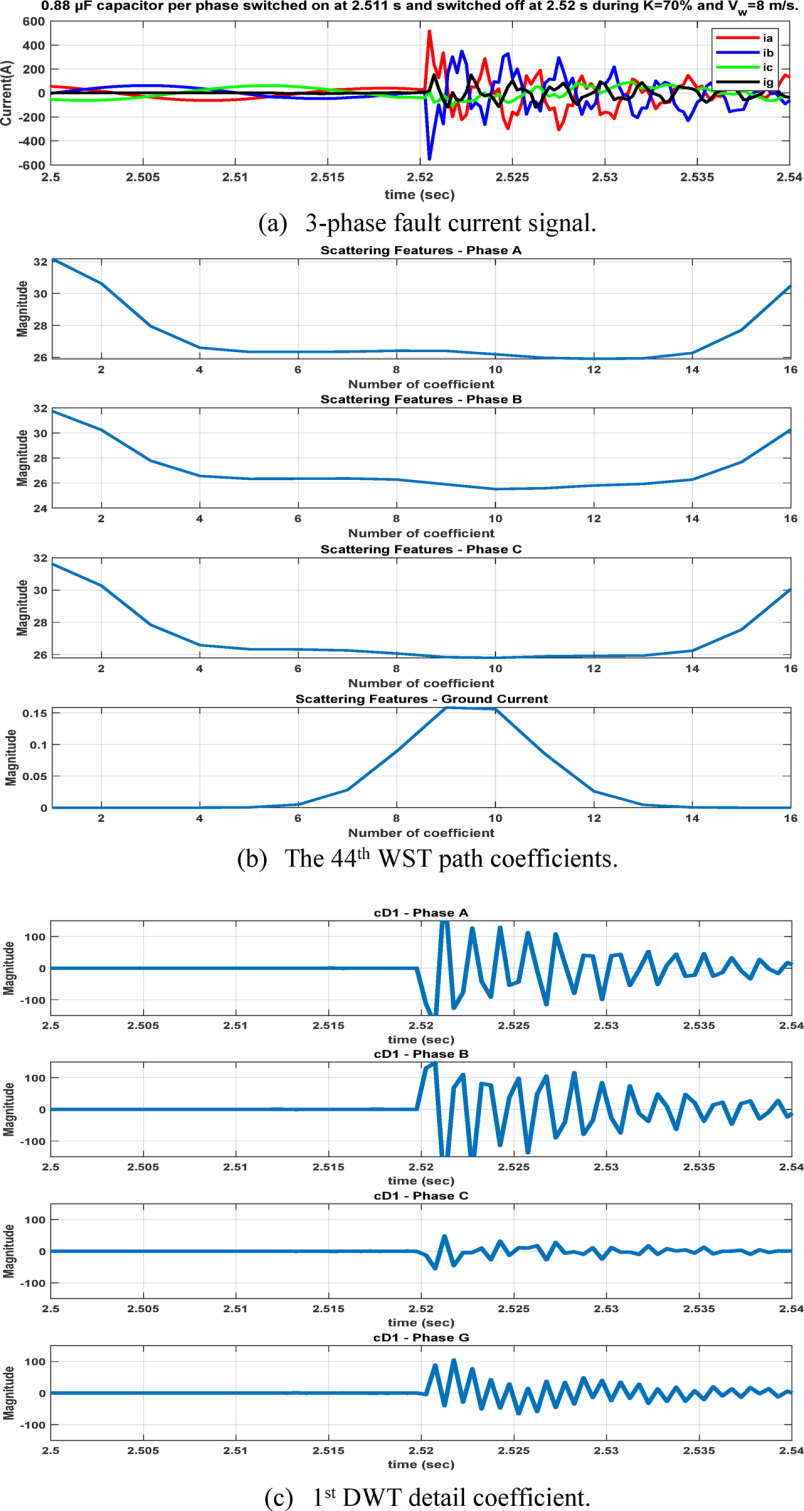

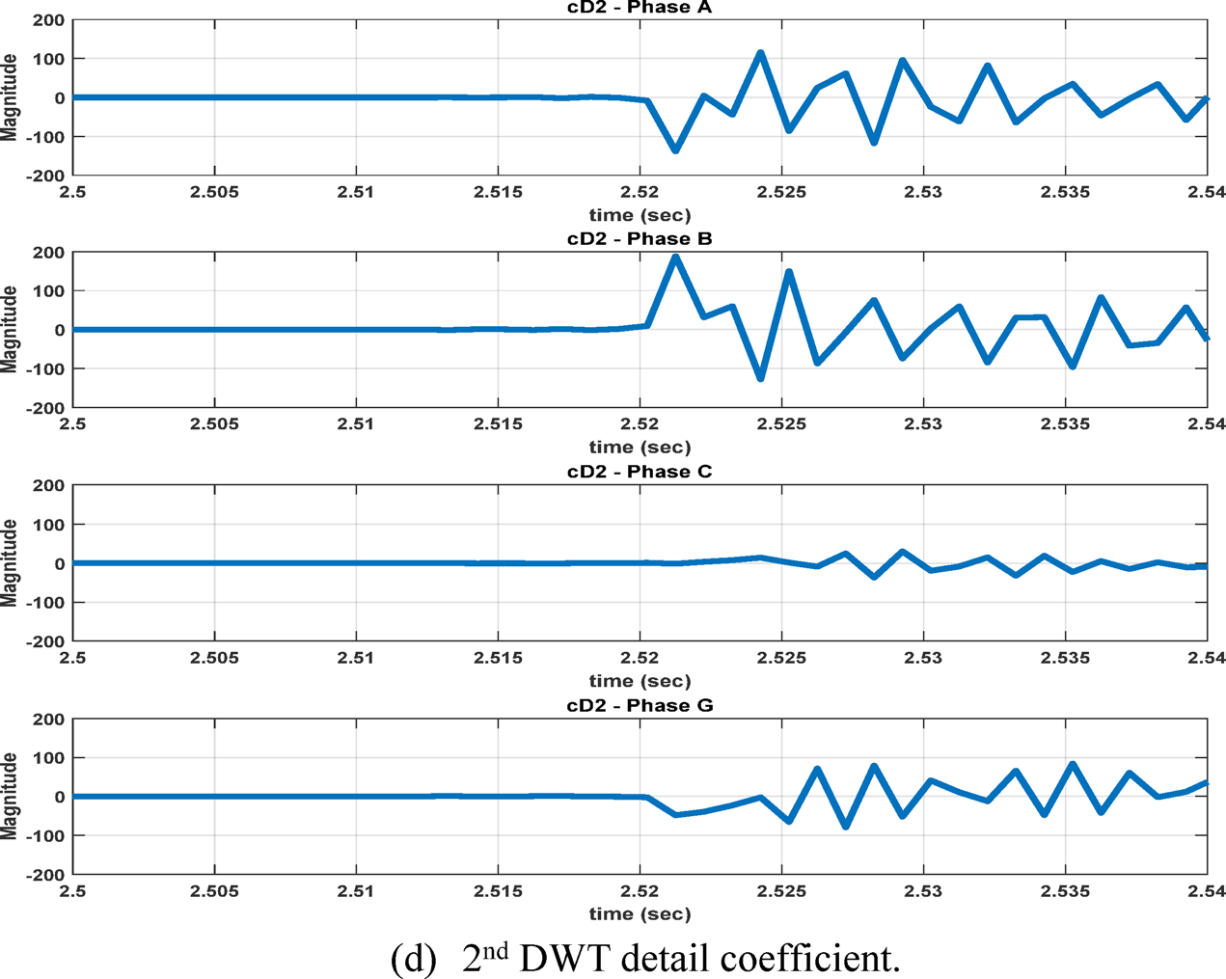
The four measured signals, their WST and DWT coefficients during per phase 0.88 $\mu F$ capacitor switching on at 2.511s and switch off at 2.52 during K = 70% and 8 m/sec wind speed.

**Fig. 24:**
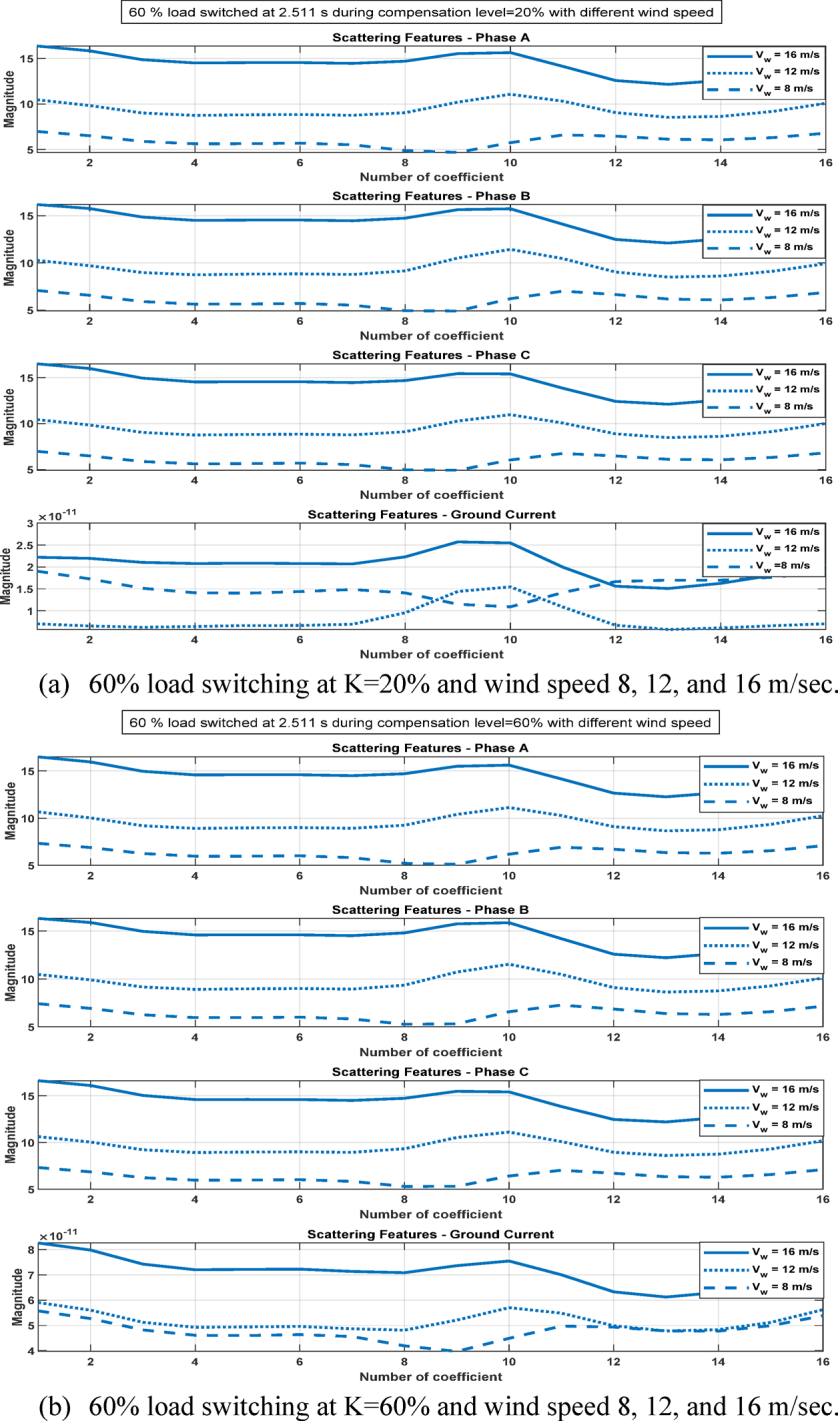
The 44th WST path coefficients of the four measured signal during 60% load switching at different compensation factor and different wind speed.

Accordingly, this superior method is further compared with several state-of-the-art external methods reported in the literature, as presented in Table 10, to highlight its effectiveness and competitiveness for fault classification applications. Moreover, the WST-based approach provides a great superiority for classification of switching operation unlike DWT based approach as in Figures 22 and 23. The WST coefficients of the four measured signals during a 60% load switching event, under different compensation levels and wind speed conditions, are illustrated in Figure 24 to demonstrate the stability of the proposed scheme under varying operating conditions. The WST coefficients exhibit relatively low magnitudes that remain distinct from those associated with fault conditions, indicating the ability of the proposed method to effectively discriminate between switching events and actual faults.

**Table 10:** Comparative assessment result.

References	Methodology	Objective	Measured data	Fundamental frequency (Hz)–Sampling frequency (kHz)	Network construction	FD accuracy %	FC accuracy %
^2^	Travelling wave	Fault location	Voltage and Current	50–5	TCSC- based compensated TL	–	–
^29^	Sign of + ve sequence current, EMD, random forest	FD, FC	Current	50–1	TCSC- based compensated TL—wind farm	100	99.85
^30^	Differential based protective relay, support vector machine	FD	Voltage and Current	60–10	Wind farm	–	–
^31^	WT, travelling wave, probabilistic neural network	FC, Fault location	Voltage and Current	50–50	TCSC- based compensated TL	–	98
^32^	S-transform, BPNN	FD, FC	Current	50–1	TCSC- based compensated TL—wind farm	100	99.99
Proposed method	WST, BPNN	FD, FC	Current	50–4	TCSC- based compensated TL—wind farm	100	100

**Fig. 25:**
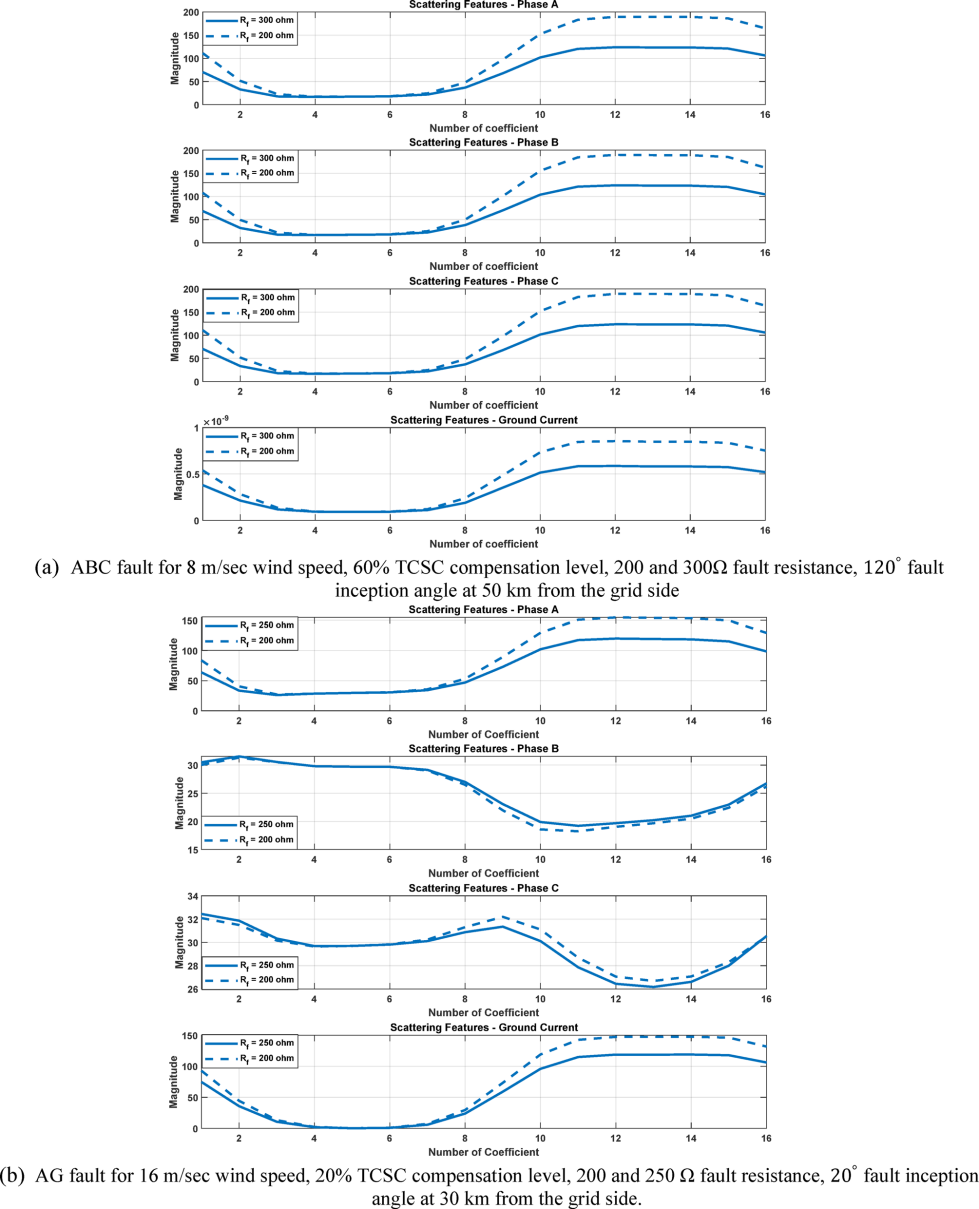
The 44th WST path coefficients using 4096 samples, [8 - 1] quality factor and 4000 Hz sampling rate.

Simulation results for high-impedance faults with resistances of 200 Ω and 300 Ω were conducted in order to further assess the effectiveness of the suggested classifier in challenging conditions. The distinguishable of fault features may be impacted by high-impedance faults, which usually result in lower fault currents and weaker transient signatures. The suggested approach based on the WST exhibits dependable classification performance in spite of these difficulties, see Figure 25. Even in situations with considerable fault resistance, the features extracted are still sufficiently discriminative to allow for precise fault type determination (notice the WST coefficient magnitude). These findings validate the suggested classifier’s resilience in managing fault scenarios with both low and high impedance.

**Fig. 26:**
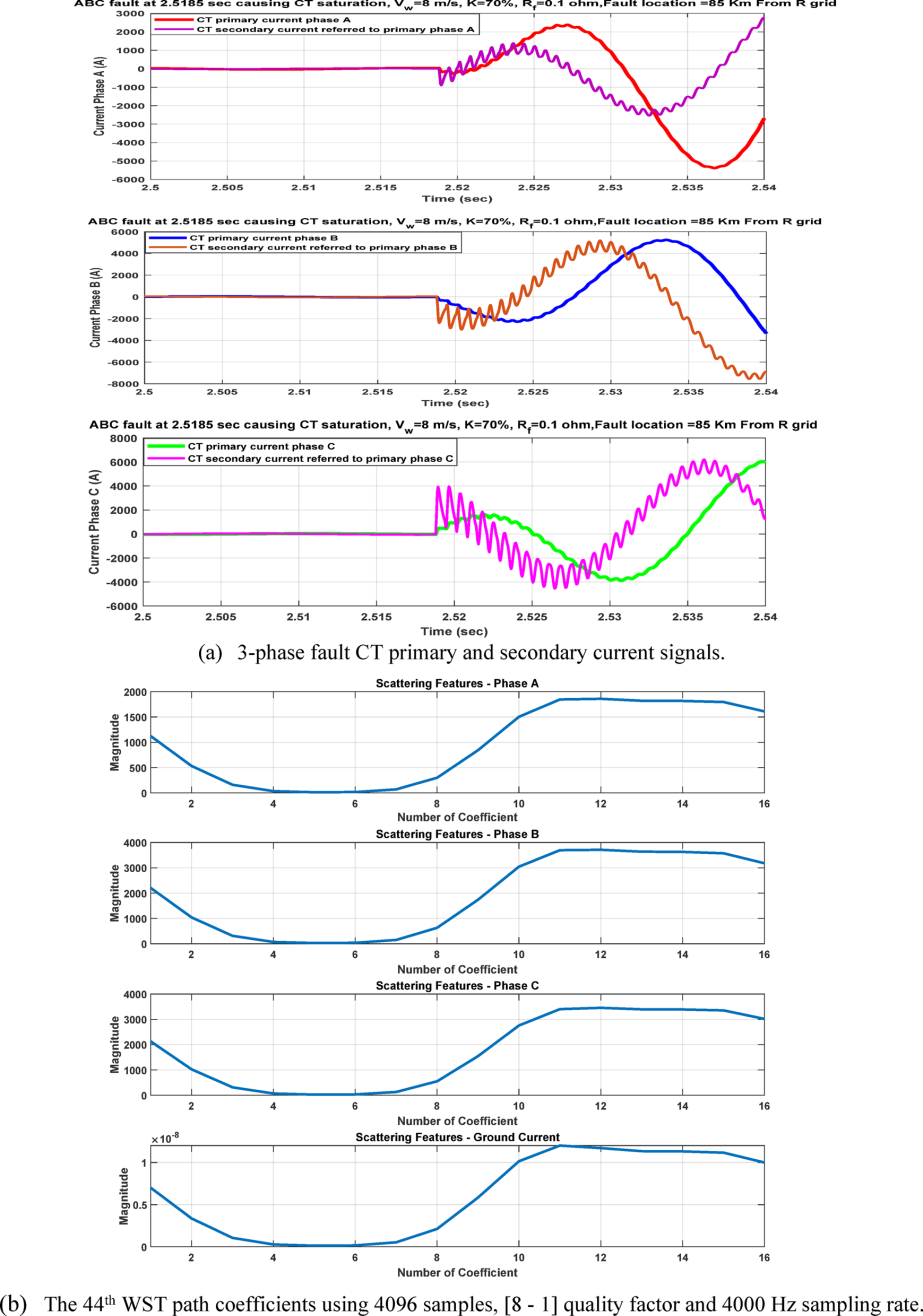

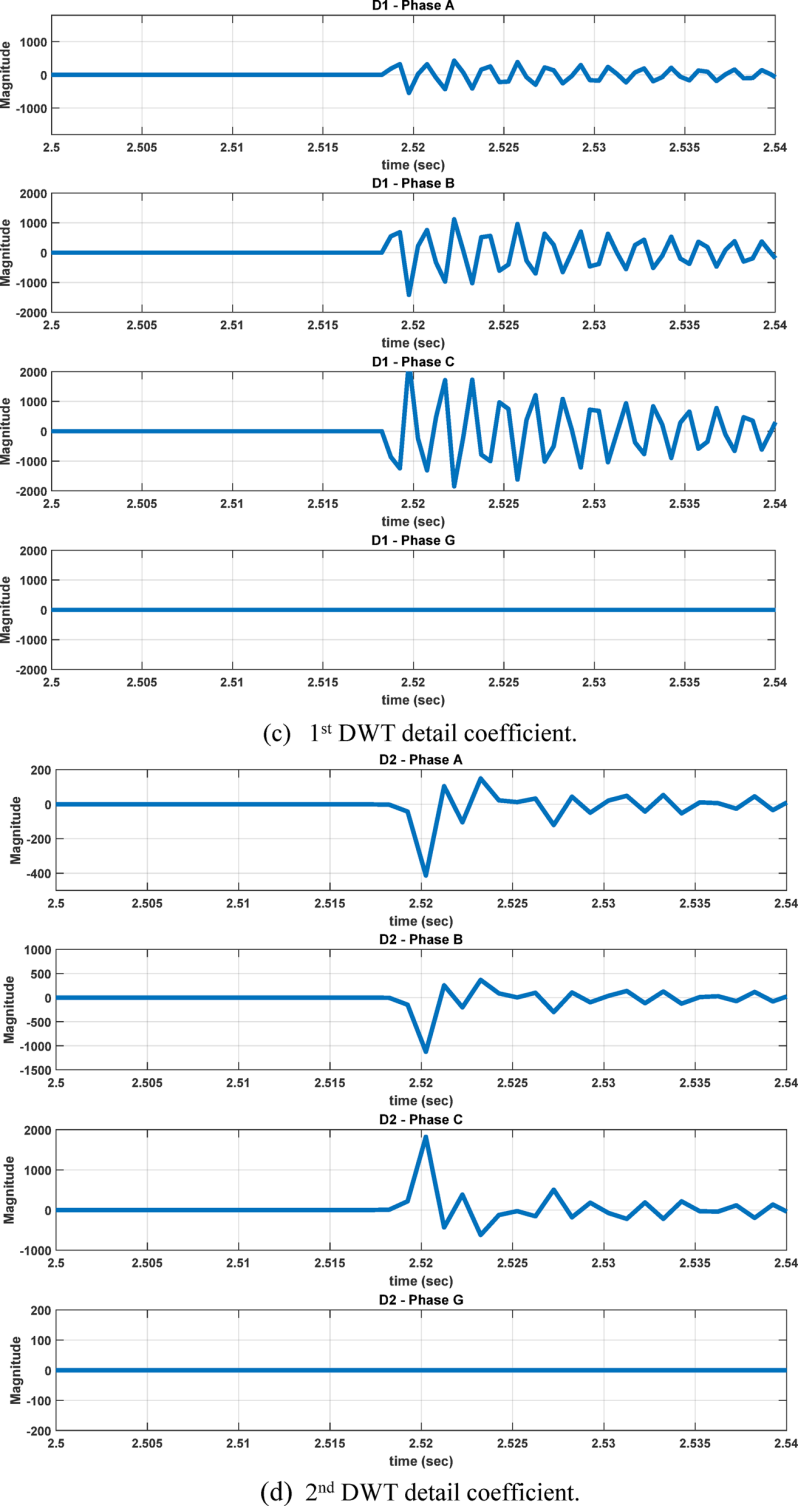
The four measured signals, their WST and DWT coefficients during ABC fault at 2.5185s causing CT saturation for wind speed 8m/sec, fault resistance 0.1Ω, and 70% compensation factor, fault location 85 km from the grid side.

**Fig. 27:**
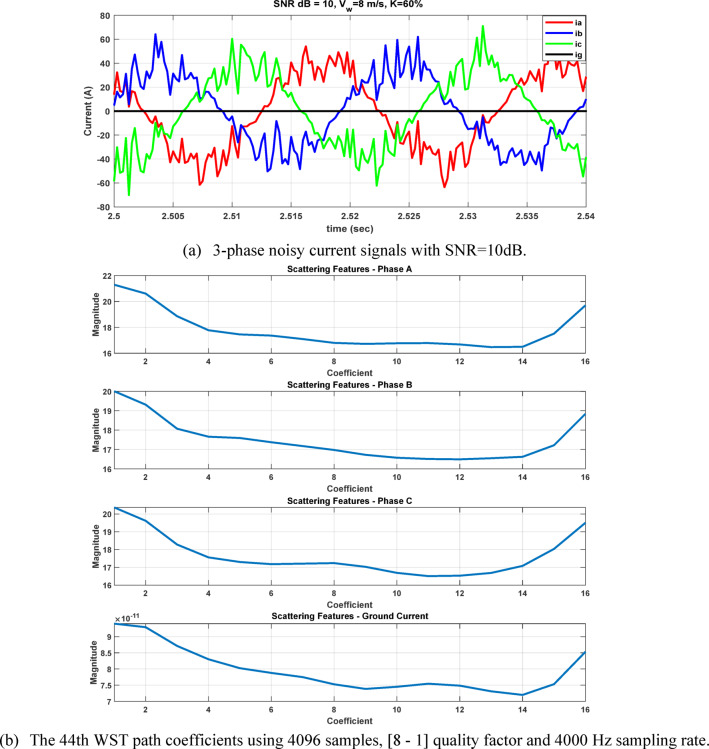

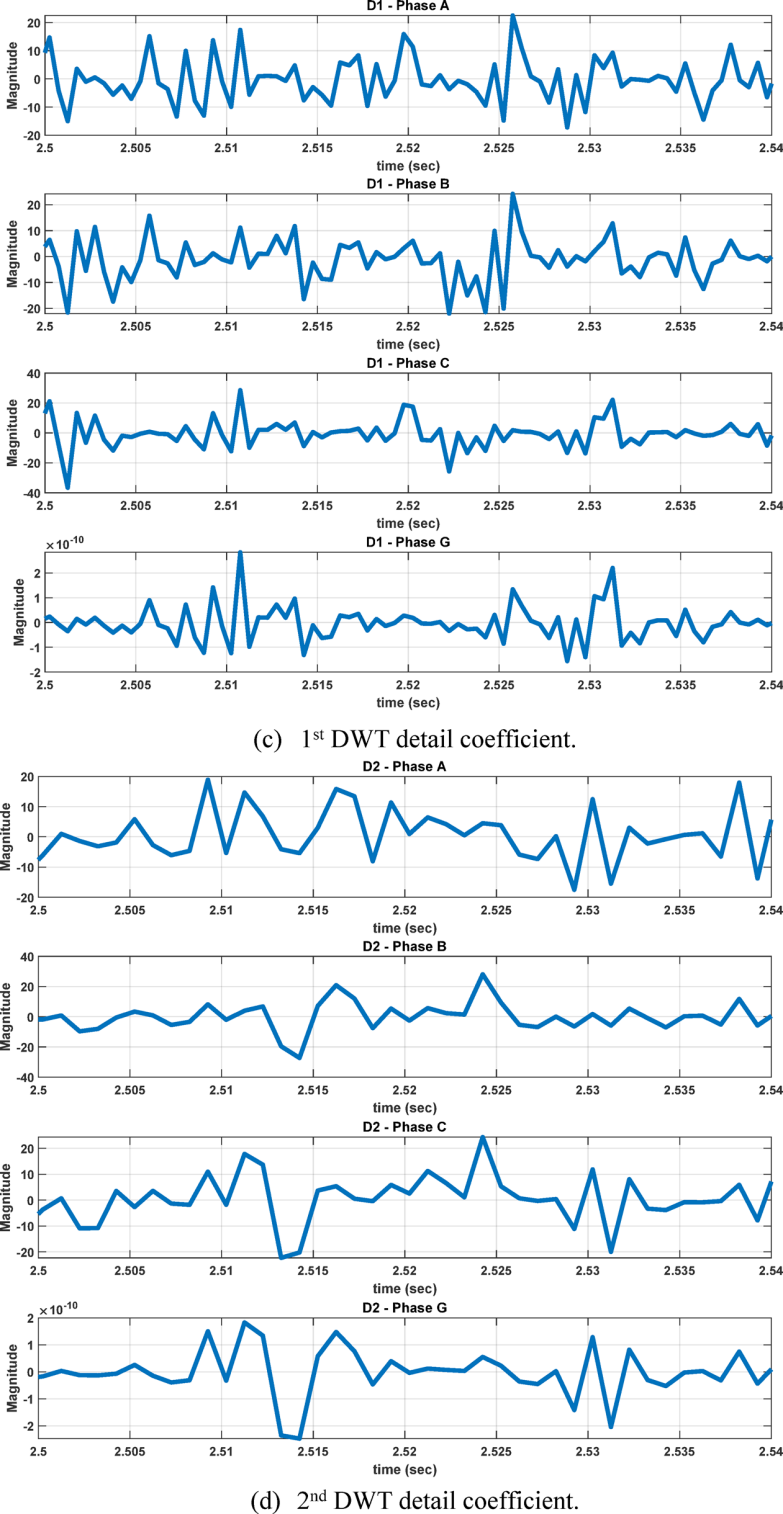

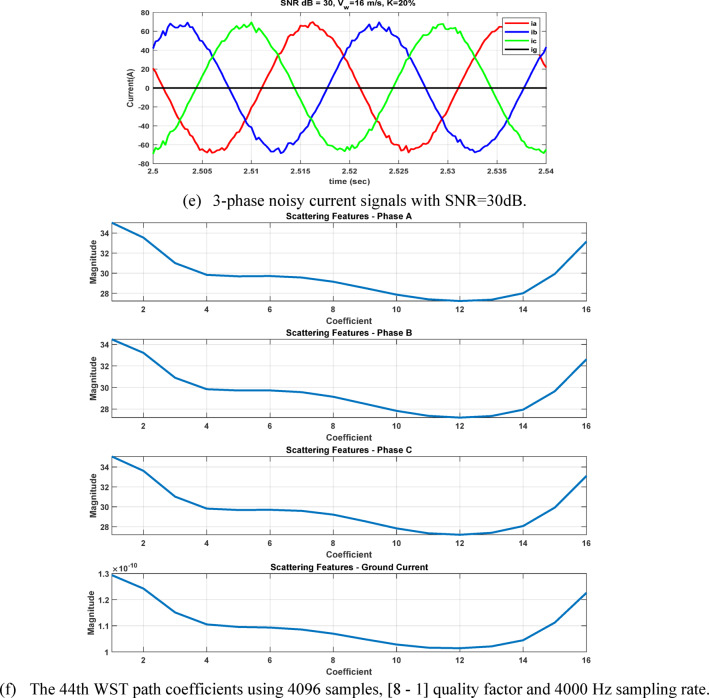

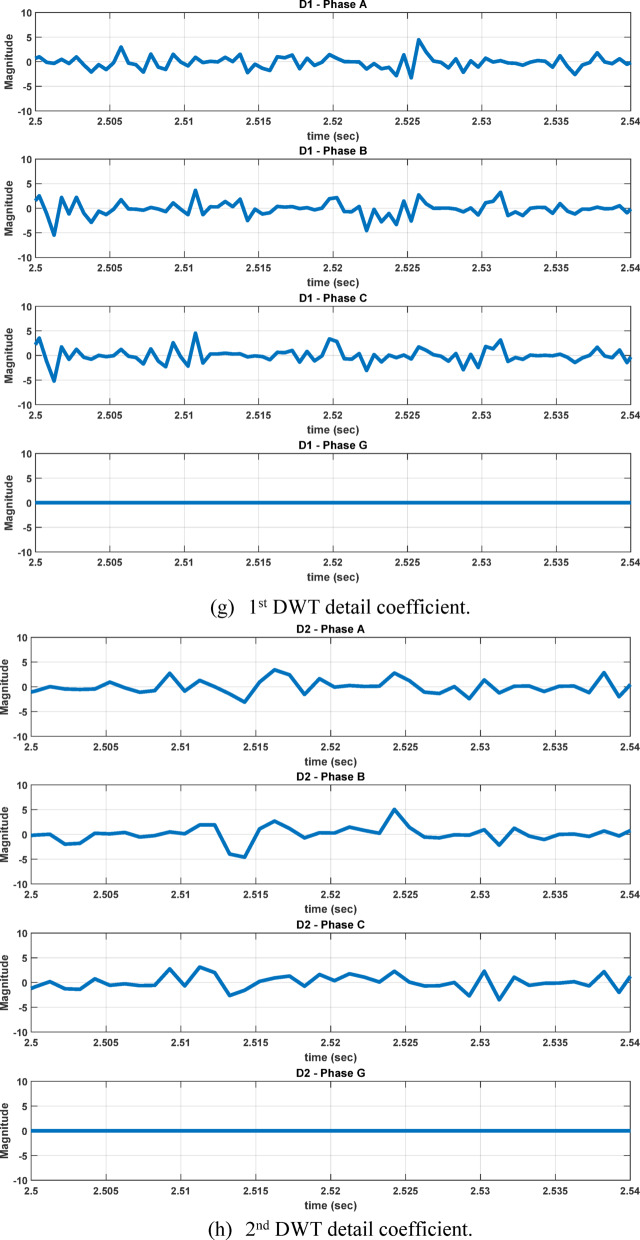
The four measured signals, their WST and DWT coefficients with SNR = 10 and 30dB.

**Fig. 28:**
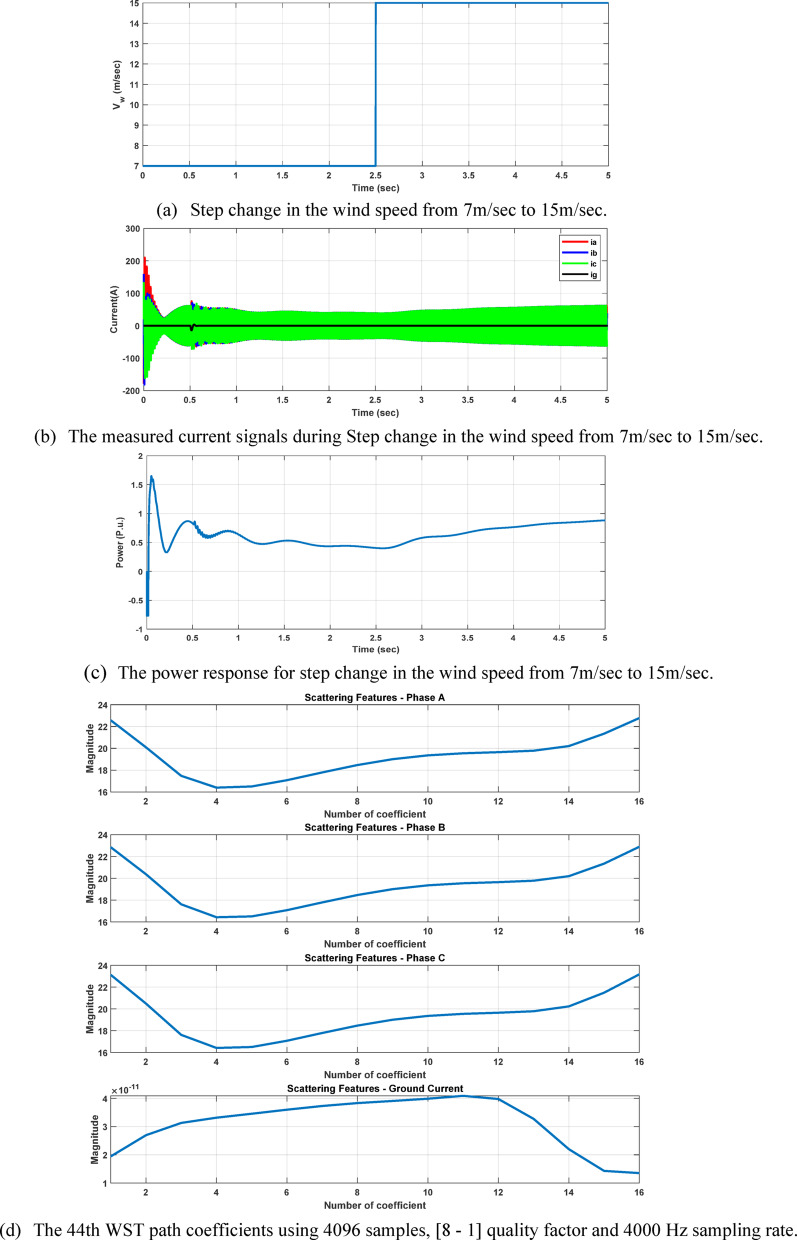
Step change in wind speed from 10 m/sec to 15 m/sec.

To evaluate the robustness of our approach under practical conditions, the impacts of current transformer (CT) saturation and measurement noise were considered. Signal-to-Noise Ratio (SNR) of 10 dB and 30 dB were applied to the measured signals to represent ideal, moderate, and severe noise environments, respectively. The results demonstrate that the proposed classifier maintains high accuracy even under distorted and noisy conditions, see Figures 26 and 27. The case shown in Fig. 28, which considers a wind speed step change from 7 to 15 m/sec, represents a practical scenario of wind-farm dynamic uncertainty, as it reflects a sudden variation in the operating conditions of the DFIG-based generation system. The results confirm that the proposed method maintains reliable performance under this type of dynamic variation.

## Conclusion

In this study an intelligent fault detection and classification scheme for TCSC-based compensated transmission lines connected to a DFIG-based wind farm has investigated addressing the challenges introduced by renewable integration and series compensation. By combining the WST for feature extraction with a BPNN for classification, the proposed approach effectively captures fault-related features from transmission line current signals under highly dynamic operating conditions.

Comprehensive simulation studies have been conducted using 3240 fault cases for training, and an additional other completely different 480 fault cases for testing covering a wide range of scenarios including different fault types, locations, inception angles, wind speeds, compensation levels, and switching events. The obtained results demonstrate that the WST-based features exhibit strong stability and discriminative capability, showing insensitivity to fault inception angle and system parameter variations. The proposed WST–BPNN scheme achieves high classification accuracy up to 100% across all evaluated scenarios, while maintaining a computational response time suitable for practical protection applications. In addition, comparative analysis with DWT-based approaches confirms the superior robustness and reliability of the proposed method, particularly under noise, dynamic operating conditions, and system uncertainties.

The results of this study indicate that the proposed WST-based intelligent classification approach provides a reliable and efficient solution for modern transmission line protection and represents a promising candidate for next-generation protection schemes in series-compensated systems with high penetration of DFIG-based wind generation.

## Data Availability

The datasets used and analyzed during the current study are available from the corresponding author upon reasonable request.

## List of symbols

X_LT_: Inductive reactance of TCSC
X_CT_: Capacitive reactance of TCSC
*α*: Firing angle
*k*: Compensation level
v_w_: Wind speed
cD1: DWT first-level detail coefficients
cD2: DWT second-level detail coefficients
*C*_*D*__1__*max*_: Maximum feature value of the first-level detail coefficients
*φ*: Low-pass scaling
*S*_0_: Zero order scattering coefficients
*S*_1_: The first order scattering coefficients
*S*_2_: The second order scattering coefficients
*f*(*t*): Input signal
ψ_1__,n_: The first CWT filter bank
ψ_2__,m_: The second CWT filter bank
